# Mendelian randomization analysis reveals causal relationships between circulating cell traits and renal disorders

**DOI:** 10.3389/fmed.2024.1360868

**Published:** 2024-05-17

**Authors:** Xing-yu Shi, Qian-kun Zhang, Jie Li, Chao-yong Zhu, Lie Jin, Shipei Fan

**Affiliations:** ^1^Department of Nephrology, Lishui Municipal Central Hospital, The Fifth Affiliated Hospital of Wenzhou Medical University, Lishui, China; ^2^Department of Ophthalmology, Lishui Municipal Central Hospital, The Fifth Affiliated Hospital of Wenzhou Medical University, Lishui, China

**Keywords:** circulating cell traits, renal disorders, Mendelian randomization, genome wide association study (GWAS), causal relationship

## Abstract

**Purpose:**

The aim of this study was to investigate the causal relationships between circulating cell traits and risk of renal disorders.

**Methods:**

We applied a comprehensive two-sample Mendelian randomization (MR) analysis. Single nucleotide polymorphisms (SNPs) from publicly available genome-wide association studies (GWAS) databases were utilized. Genetically predicted instrumental variables of human blood cell traits were extracted from Blood Cell Consortium (BCX) while data on renal diseases was obtained from Finngen consortium. The primary MR analysis was conducted using the inverse variance weighted (IVW) method, with the weighted median (WM) and MR-Egger models used as additional methods. Sensitivity analyses, including MR-PRESSO, radial regression and MR-Egger intercept were conducted to detect outliers and assess horizontal pleiotropy. We further utilized the leave-one-out analysis to assess the robustness of the results. Causal associations were considered significant based on false rate correction (FDR), specifically when the IVW method provided a *p*_FDR_ < 0.05.

**Results:**

Our results demonstrated that both white blood cell (WBC) count (OR = 1.50, 95% CI = 1.10–2.06, *p*_FDR_ = 0.033, *p*_IVW_ = 0.011) and lymphocyte count (OR = 1.50, 95% CI = 1.13–1.98, *p*_FDR_ = 0.027, *p*_IVW_ = 0.005) were causally associated with a higher risk of IgA nephropathy. Furthermore, WBC count was identified as a significant genetic risk factor for renal malignant neoplasms (OR = 1.23, 95% CI = 1.06–1.43, *p*_FDR_ = 0.041, *p*_IVW_ = 0.007). Additionally, an increased level of genetically predicted eosinophils was found to be causally associated with a higher risk of diabetic nephropathy (OR = 1.21, 95% CI = 1.08–1.36, *p*_FDR_ = 0.007, *p*_IVW_ = 0.001). No evidence of pleiotropy was determined.

**Conclusion:**

Our findings provide evidence of causal associations of circulating WBC count, lymphocyte count and IgA nephropathy, WBC count and renal malignant neoplasms, and eosinophil count and diabetic nephropathy. These results have the potential to contribute to the development of novel diagnostic options and therapeutic strategies for renal disorders.

## Introduction

Chronic kidney disease (CKD) is a prevalent renal disorder characterized by abnormal kidney structure and function (1). As previously reported, CKD has a global prevalence of approximately 13% (11.7–15.1%) in adults, resulting in a significant health and economic burden (2). IgA nephropathy, characterized by the presence of IgA immune complex deposits in the glomerular mesangium, is one of the leading causes of CKD worldwide (3). Diabetic nephropathy is the primary cause of end-stage renal disease and affects approximately 30–40% diabetes individuals (4). In addition, hypertensive nephropathy is considered as the consequence of uncontrolled blood pressure, which is also identified as an essential cause of CKD (5). Renal malignant neoplasms, such as the renal cell carcinoma, share closely related molecular mechanisms with CKD (6). As epidemiological study described, the number of renal malignant neoplasms increases rapidly each year (7). However, at the early stages of these renal diseases, obvious clinical symptoms are absent. Therefore, there is an urgent need for novel and convenient biomarkers to facilitate early diagnosis and treatment options.

White blood cells (WBCs) are routinely measured in daily clinical practice and are crucial components of immune system (8). The WBCs are divided into five subgroups: neutrophils, lymphocytes, monocytes, basophils and eosinophils. Given the easy availability of WBCs and subtypes, numerous studies have investigated the associations between these circulating cells and renal diseases in recent years. A meta-analysis has indicated that the platelet-lymphocyte ratio is a reliable predictor of CKD patients and is associated with an increased risk of mortality (9). Wang et al. (10) demonstrated that the neutrophil-lymphocyte ratio (NLR) could be a sensitive biomarker of progression of renal function and prognosis of IgA nephropathy patients. Scholars from China further discovered that NLR was positively correlated with the degree of renal tubular atrophy and interstitial fibrosis (11). Additionally, a cross-sectional study has found a close association between the NLR and diabetic kidney disease, suggesting that it may serve as an effective indicator (12). However, the existing evidence is predominantly derived from observational studies, which may be susceptible to limitations such as reverse causality and confounding factors. As a result, there is a scarcity of robust evidence concerning the causal relationships between circulating cell characteristics and kidney diseases.

Mendelian randomization (MR) analysis is a novel method that employs genetic variants to infer causal relationships between risk factors and disorders. MR analysis has the ability to minimize confounding factors and exclude reverse causality by virtue of genetic variations being randomly allocated at conception. Compared to traditional clinical randomized controlled trials, MR studies offer causal inference supported by genetic evidence and avoid the effects of latent confounders. In order to investigate the potential pathogenesis of renal diseases, MR analysis was employed to elucidate the causal inference of blood cell traits on the risk of renal disorders. Exploring and understanding the risk factors associated with renal diseases will contribute novel insights to these escalating public health burdens.

## Methods

### Study design

We systematically assess the causal estimates of peripheral cell traits on the risk of kidney diseases using comprehensive two-sample MR analyses. The present MR study follows the strengthening the reporting of observational studies in epidemiology using Mendelian randomization (STROBE-MR) guideline (13). We performed MR analyses to estimate causal associations between circulating cell traits and renal diseases, using publicly available genome-wide association studies (GWAS) statistics. To obtain reliable data, the MR analyses were performed in accordance with three essential assumptions: (1) the instrumental variables (IVs) should be closely and robustly associated with circulating blood cell traits; (2) the genetic variants and outcomes are independent each other; (3) the IVs are not related with any potential confounding factors.

### Exposure sources of circulating cell traits

Six phenotypes of circulating cell traits, including the level of white blood cell (WBC), neutrophil, lymphocyte, monocyte, basophil and eosinophil counts were recruited as exposure variants. Instrumental genetic variables for these cell traits were extracted form Blood Cell Consortium (BCX, 563,085 European ancestry participants) (14). To ensure strong correlations between SNPs and exposure, we extracted the exposure SNPs with genome-wide significance (*p* < 5 × 10^−8^). Linkage disequilibrium analysis (*r*^2^ < 0.001, genome region = 10,000 kb) was performed to align with the MR assumptions. The *F* value was determined to assess the instrument strength using the formula (*F* = Beta^2^/SE^2^) (15). To avoid bias caused by weak instrumental variables, SNPs with a *F* value less than 10 were eliminated. Palindromic and incompatible genetic variables were selected and subsequently discarded to ensure the robustness of statistics.

### Outcome sources of renal disorders

Considering the outcome data, we searched summary statistics datasets from latest Finngen project,^1^ which contains 377,277 individuals and 20,175,454 variants (16). After adjusting for age, sex, genetic relatedness and genotyping batch, detailed and comprehensive genetic variables were extracted for IgA nephropathy (592 cases and 376,685 controls), renal malignant neoplasms (2,223 cases and 287,137 controls), hypertensive nephropathy (1,022 cases and 265,626 controls) and diabetic nephropathy (4,111 cases and 308,539 controls).

### Statistical analyses

**Figure 1:**
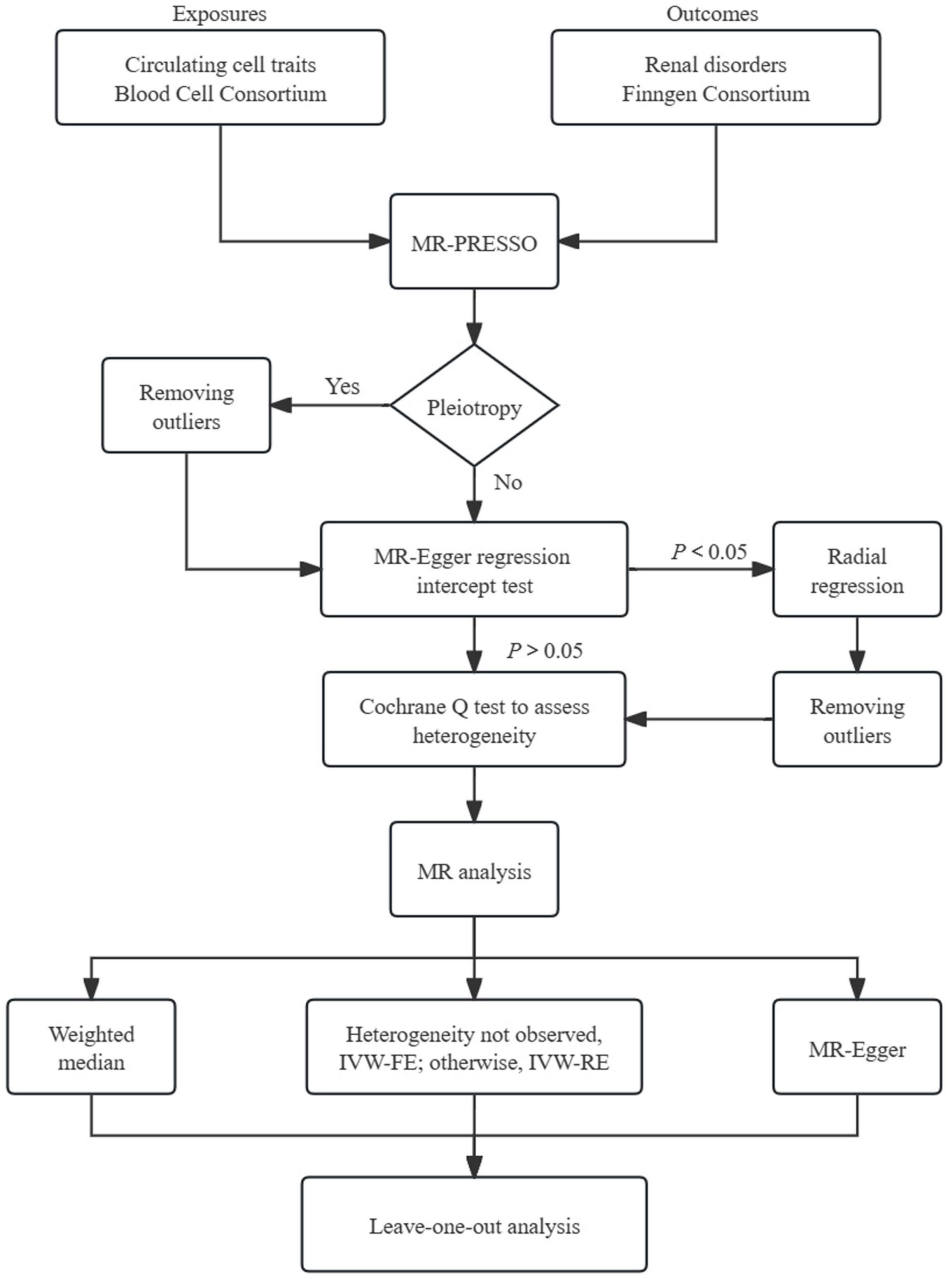
Schematic representation of Mendelian randomization analysis. FE, fixed-effect; RE, random-effect; MR-PRESSO, MR pleiotropy residual sum and outlier.

The overall design of MR analysis was illustrated in Figure 1. Initially, we conducted the MR-PRESSO global test to assess the potential horizontal pleiotropy (17). In cases where horizontal pleiotropy was detected, we identified and eliminated outliers. Subsequently, we employed the intercept of MR-Egger regression to assess the horizontal pleiotropy (18). If statistically significant pleiotropy was identified through the MR-Egger intercept test, we adopted radial regression to further evaluate and exclude outliers (19).

The inverse variance weighted (IVW) analysis, which incorporates information from all instrumental variables (IVs), is widely recognized for providing precise and robust results (20). We performed the fixed-effects IVW test as the primary approach to determine the causal association. In instances of significant heterogeneity, alternative multiplicative random-effects IVW model was used. Additionally, we employed the weighted median (WM) method and MR-Egger test as supplementary approaches. The WM method enables robust estimation of causal effects, even when up to 50% of the genetic instruments are invalid (21). The MR-Egger model is capable of identifying deviations from key assumptions of MR and generating effect estimates that are not influenced by these biases. To quantify the causal relationships, odds ratios (ORs) with their corresponding 95% confidence intervals (CIs) were used. We conducted Cochran’s *Q* test to assess the heterogeneity. Additionally, we performed the leave-one-out analysis to further assess the robustness of results.

All statistical analyses were performed using R software (Version 4.3.1). The R packages “TwoSampleMR” and “MR-PRESSO” were adopted to assess causal estimates between cell traits and kidney diseases. We applied a threshold of 0.05 for the false discovery rate correction (FDR). Causal relationships were deemed statistically significant when a value of *p*_FDR_ < 0.05. In cases where the *p*-value from the IVW method was less than 0.05 but the *p*_FDR_ was greater than 0.05, a suggestive causal link was indicated.

## Results

Firstly, we utilized the MR-PRESSO test to detect horizontal pleiotropy and eliminate outliers. No outliers were observed among the genetic variables in IgA nephropathy, renal malignant neoplasms, and hypertensive nephropathy. However, horizontal pleiotropy was detected for the lymphocyte count in IgA nephropathy using the intercept of MR-Egger. Furthermore, radial regression was performed, resulting in the removal of 31 SNPs. Considering the diabetic nephropathy, outliers of basophil (rs3184504), WBC (rs3184504, rs72982988), monocyte (rs1146933) and eosinophil counts (rs4652560) were identified and excluded using the MR-PRESSO method. Horizontal pleiotropy was detected when WBC and neutrophil counts were exposures. To ensure the reliability of results, the radial regression was adopted, which led to further exclusion of 37 SNPs for WBC count and 38 SNPs for neutrophil count.

After harmonizing and selecting of IVs from the blood cell traits and GWAS datasets of renal disorders, we utilized 185–470 associated SNPs for the six cell traits. The F statistics of all included SNPs were above 10 (ranged from 29.8 to 4558.5), indicating that sufficiently strong genetic instruments were obtained in the present MR analysis. The detailed characteristics including the rs. number, effect allele, other allele, beta value, standard error and effect allele frequency, were listed in Supplementary Table S1.

**Figure 2:**
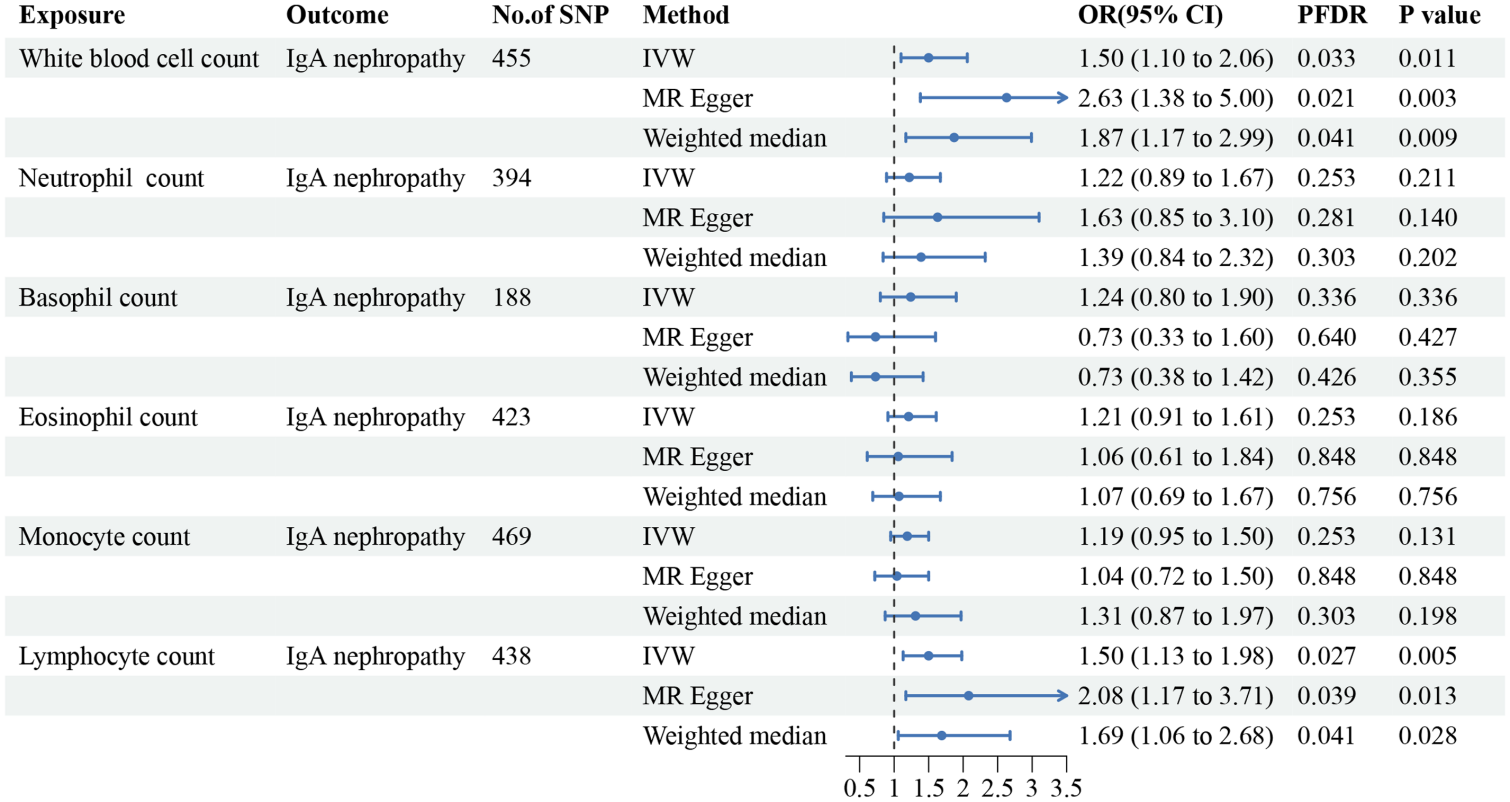
Forest plot for the causal estimates of circulating cell traits on the risk of IgA nephropathy. SNP, single nucleotide polymorphism; IVW, inverse variance weighted; OR, odds ratio; 95% CI, 95% confidence interval; PFDR, *p*-value after false discovery rate correction.

**Figure 3:**
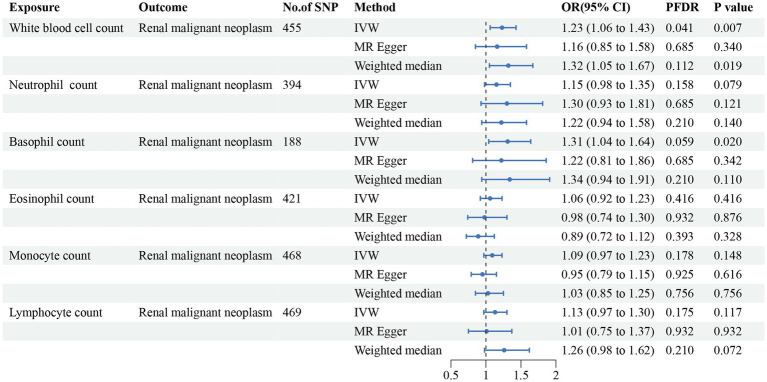
Forest plot for the causal estimates of circulating cell traits on the risk of renal malignant neoplasms. SNP, single nucleotide polymorphism; IVW, inverse variance weighted; OR, odds ratio; 95% CI, 95% confidence interval; PFDR, *p*-value after false discovery rate correction.

**Figure 4:**
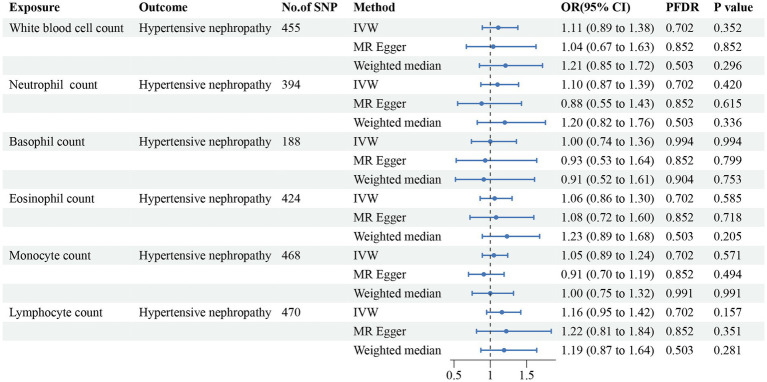
Forest plot for the causal estimates of circulating cell traits on the risk of hypertensive nephropathy. SNP, single nucleotide polymorphism; IVW, inverse variance weighted; OR, odds ratio; 95% CI, 95% confidence interval; PFDR, *p*-value after false discovery rate correction.

**Figure 5:**
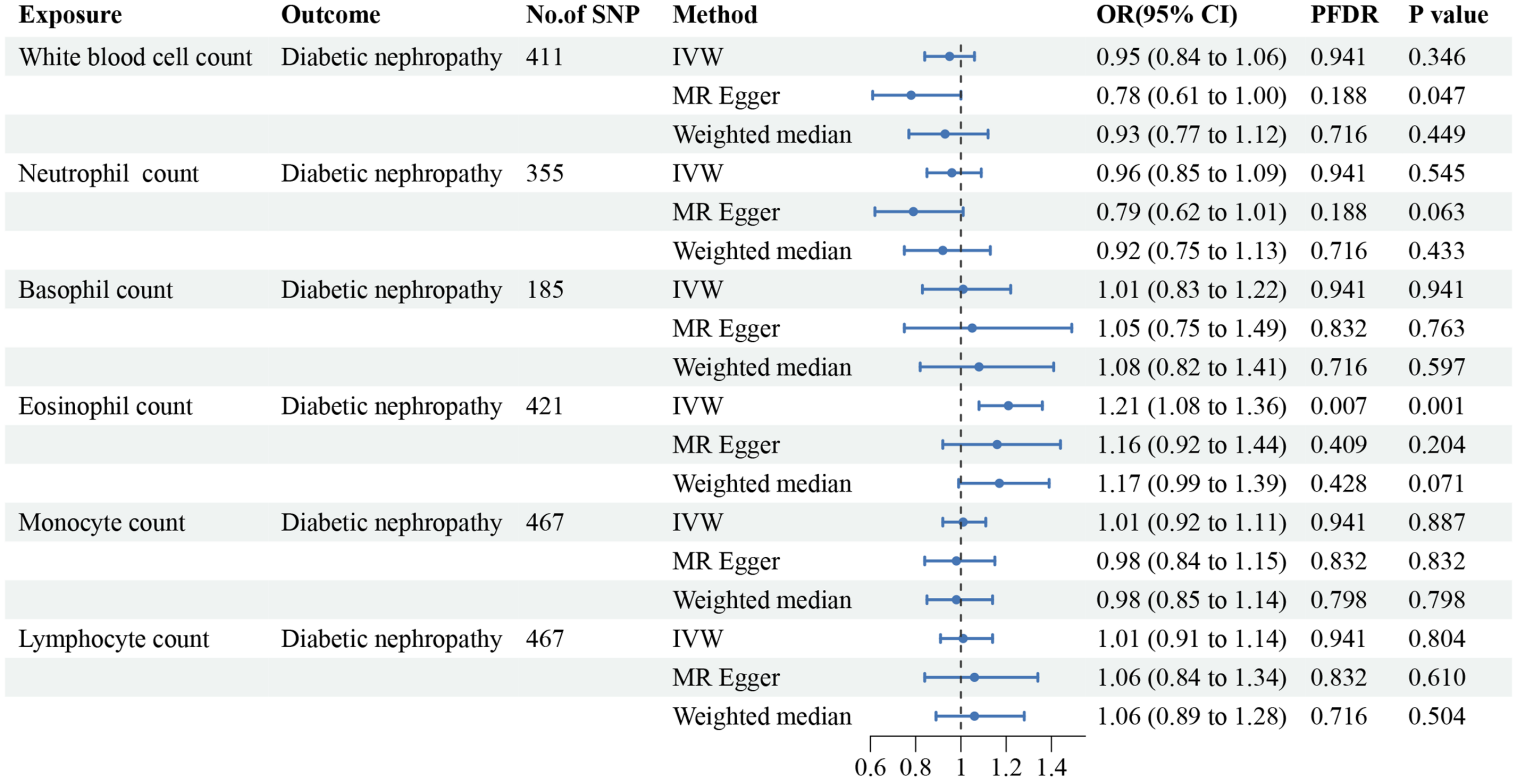
Forest plot for the causal estimates of circulating cell traits on the risk of diabetic nephropathy. SNP, single nucleotide polymorphism; IVW, inverse variance weighted; OR, odds ratio; 95% CI, 95% confidence interval; PFDR, *p*-value after false discovery rate correction.

The causal links and effects are summarized in Figures 2–5. Regarding IgA nephropathy, we confirmed that increased WBC count (OR = 1.50, 95% CI = 1.10–2.06, *p*_IVW_ = 0.011) and lymphocyte count (OR = 1.50, 95% CI = 1.13–1.98, *p*_IVW_ = 0.005) were positively associated with a higher risk of IgA nephropathy. These associations remained statistically significant after FDR correction (*p*_FDR_ = 0.033 and 0.027 respectively). Consistent results were observed with MR-Egger and WM methods (*p*_FDR_ < 0.05). No obvious relationships or suggestive links were found between neutrophil, basophil, eosinophil or monocyte counts and IgA nephropathy susceptibility.

**Figure 6:**
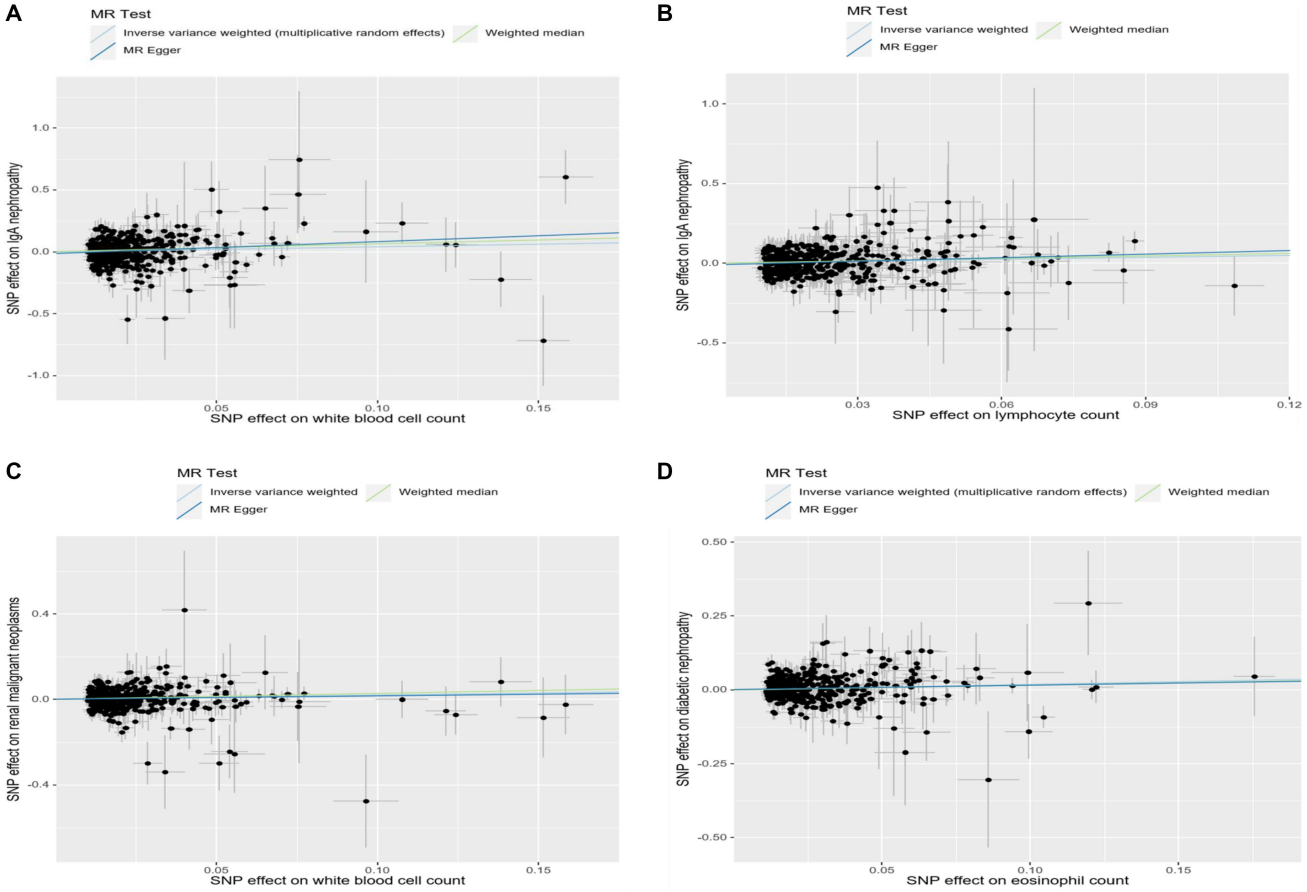
Scatter plots showed the effect size of each single-nucleotide polymorphism (SNP) on exposures and outcomes. **(A)** Scatter plot of SNP-white blood cell count and SNP-IgA nephropathy association. **(B)** Scatter plot of SNP-lymphocyte count and SNP-IgA nephropathy association. **(C)** Scatter plot of SNP-white blood cell count and SNP-renal malignant neoplasms association. **(D)** Scatter plot of SNP-eosinophil count and SNP-diabetic nephropathy association.

In addition, we found that genetically predicted WBC count had a risk effect on renal malignant neoplasms (OR = 1.23, 95% CI = 1.06–1.43, *p*_FDR_ = 0.041, *p*_IVW_ = 0.007). Similar trends were determined using additional models, without statistical significance. Basophil count was also potentially associated with an elevated risk of renal malignant neoplasms using the IVW method (OR = 1.31, 95% CI = 1.04–1.64, *p*_IVW_ = 0.020), indicating a suggestive relationship after FDR correction (*p*_FDR_ = 0.059). Considering hypertensive nephropathy, no significant or suggestive associations were confirmed between the cell traits and outcome. Notably, we observed that an increased genetically assessed eosinophil count was significantly associated with a higher risk of diabetic nephropathy (OR = 1.21, 95% CI = 1.08–1.36, *p*_FDR_ = 0.007, *p*_IVW_ = 0.001). The causal estimates from MR-Egger and WM methods demonstrated similar trends. We found no obvious relationships between other five cell traits and diabetic nephropathy. To enhance the visualization, scatter plots were utilized to depict the effects of SNPs on cell traits and four renal disorders (Figure 6; Supplementary Figures S1–S20).

To evaluate the reliability and stability of the MR results, we performed sensitivity analyses. We applied Cochran’s *Q* test of IVW model to assess the heterogeneity, which was summarized in Supplementary Table S2. The MR-PRESSO method and radial MR regression were employed to identify potential outliers and further removed. As shown in Supplementary Table S2, no horizontal pleiotropy was determined using intercept of MR-Egger. The funnel plots revealed the distribution of the effect of a single SNP, indicating the consistency and reliability of the causal estimates in this MR analysis (Figure 7; Supplementary Figures S21–S40). Leave-one-out analyses demonstrated that removal of any SNP individually could not affect the effect of causal estimates, confirming the robustness of the results (Supplementary Table S3).

**Figure 7:**
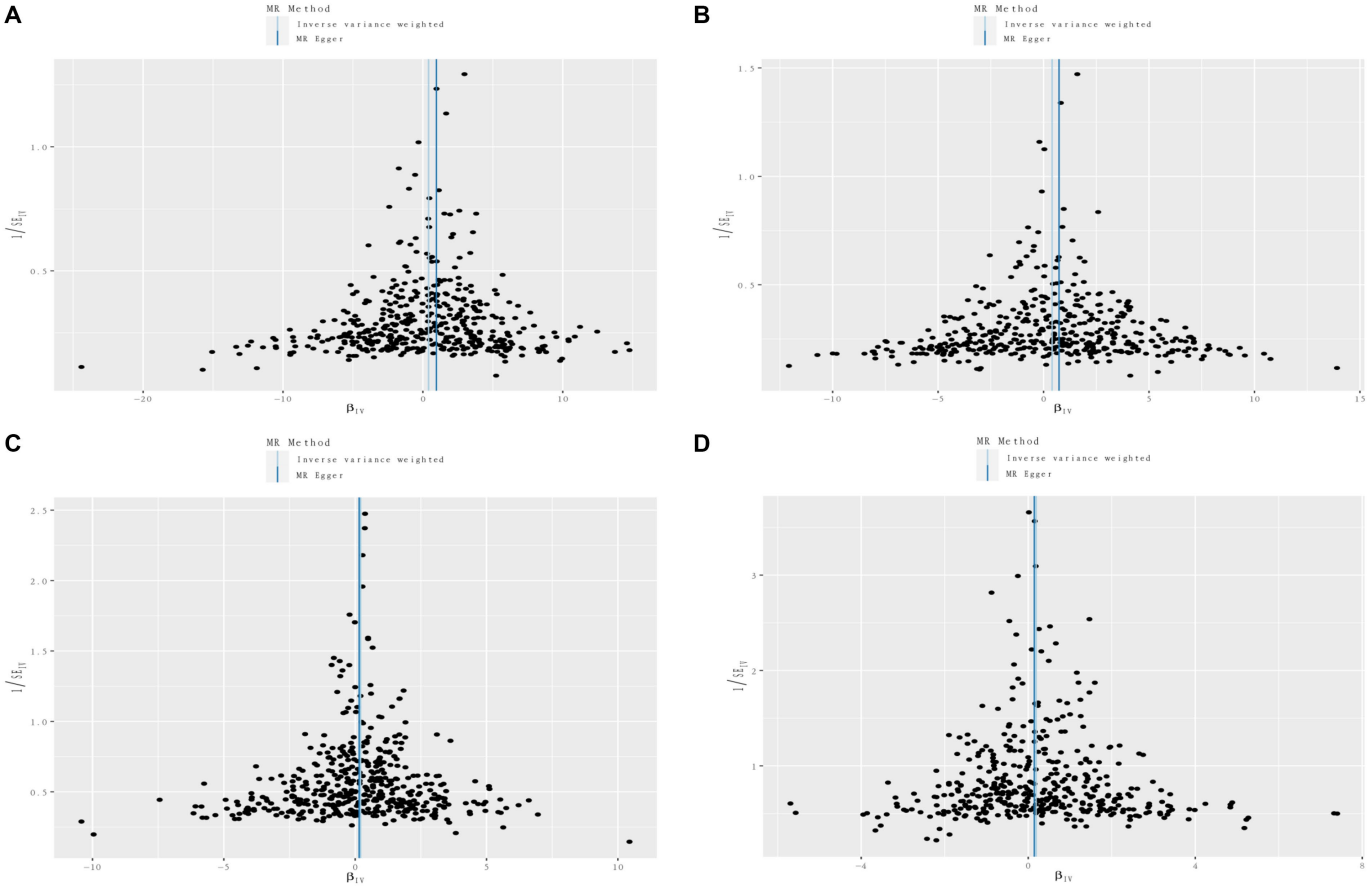
Funnel plots of circulating cell traits genetic variants and renal disorders. **(A)** Funnel plot of white blood cell count and IgA nephropathy. **(B)** Funnel plot of lymphocyte count and IgA nephropathy. **(C)** Funnel plot of white blood cell count and renal malignant neoplasms. **(D)** Funnel plot of eosinophil count and diabetic nephropathy.

## Discussion

In this study, we provide valid evidence and valuable information on the causal associations between circulating leukocyte count, lymphocyte count and IgA nephropathy, as well as leukocyte count and renal malignant neoplasms. We also demonstrate the causal relationship between eosinophil count and diabetic nephropathy from a genetic perspective. Considering the relative scarcity investigations in this field, our results provide essential insights into the control and prevention strategies for renal diseases.

Longitudinal follow-up investigations have demonstrated that approximately 20–40% IgA nephropathy patients will develop chronic renal insufficiency or end-stage renal disease (22, 23). Despite numerous publications on various aspects of this disease, the precise mechanisms remain poorly understood. However, it has been observed that leukocytic infiltration into the kidney is closely associated with glomerulonephritis and renal failure (24, 25). As prior bioinformatics analysis described, extensive upregulated genes are closely related with leucocyte migration in IgA nephropathy, which is in line with our results (26). Using MR analysis, we provide genetic evidence indicating an increased lymphocyte count is causally associated with the risk of IgA nephropathy. Given that lymphocytes make up approximately 20–30% of the WBC count, we speculate that the relationship between WBC count and IgA nephropathy is primarily due to the association between lymphocyte count and IgA nephropathy. Previous immunological study has demonstrated that elevated numbers of lymphocytes, increased percentages of activated CD8^+^ T lymphocytes and CD4^+^ T lymphocytes in IgA nephropathy patients were observed, compared to healthy individuals (27). Additionally, excessive activity of T lymphocytes has been proved to be correlated with clinical severity of IgA nephropathy (28). T lymphocytes can activate the release of pro-inflammatory cytokines and chemokines, including interleukin-1, interleukin-6 and tumor necrosis factor, leading to immune deposits and further stimulating mesangial cells, thereby accelerating renal injury and disease progression (29, 30). Thus, lymphocytes are considered to play a vital role in the occurrence and progression of IgA nephropathy.

In recent years, the MR analysis has been utilized to explore and elucidate causal inference between leukocytes and various cancer types. Previous MR analyses have reported that genetically predicted leukocyte telomere length can either increase or decrease the risk of different types of cancers, highlighting the essential role of leukocyte in cancer pathogenesis (31, 32). Elevated concentrations of LINE-1 methylation in DNA and mitochondrial DNA copy number in leukocytes were observed in renal cell carcinoma patients, indicating the complex mechanisms of leukocytes in renal cancers (33, 34). Our study reveals a significant causal relationship between increased levels of leukocyte and risk of renal malignant neoplasms, in accord with experimental investigations. Moreover, basophil count has also shown a potential association with renal malignant neoplasms. As previously described, basophils participate in secreting cytokines and inflammatory mediators, leading to abnormal tumor microenvironments (35, 36). Further explorations are necessary to understand the detailed pathogenesis.

Diabetic nephropathy, accounting for approximately 30–50% of end-stage kidney disease, is the leading cause of CKD (37). Our results have identified the eosinophil count as a significant risk factor for diabetic nephropathy. Eosinophils are now recognized as a source of inflammatory mediators and oxidative factors, participating in various immune responses and conditions. Protein expression profile study has identified upregulated levels of eosinophil cationic protein in human glomerular mesangial cells, stimulated by high glucose (38). A retrospective study conducted in the Chinese population has also found an association between eosinophils and degree of renal injury, as determined by renal biopsy (39). The researchers speculate that glycation end products, under the abnormal glucose control, lead to eosinophils aggregates and further stimulate local inflammation responses.

Several limitations should be considered in this study. Firstly, to ensure validity of the data, we adopted multiple sensitivity analyses, including intercept of MR-Egger, MR-PRESSO method, radial regression and leave-one-out analysis. However, it is impossible to completely rule out and avoid the potential pleiotropy. Therefore, causal inference should be interpreted with caution, further clinical and experimental researches are need to validate the results. Secondly, the GWAS databases used in this MR analysis are obtained from individuals of European ancestry. Taken the potential association between causality and race into account, the findings of this study may not be applicable when generalizing the causal inference into other populations. Moreover, due to the limitations of current data, we are unable to access further information such as the classifications of disorders, the status of kidney function and blood glucose, age and gender of participants. In the future, MR analyses which take these factors into consideration could provide further evidence-based guidance for investigators.

In conclusion, our MR analysis findings offer genetic evidence for causal relationships between increased peripheral cell counts and higher risks of renal disorders. These results may contribute to the exploration of potential clinical biomarkers and ultimately improve accurate diagnosis, treatment and prognosis. Further investigations are desirable to elucidate the complex pathogenesis in the future.

## Data availability statement

The original contributions presented in the study are included in the article/Supplementary material, further inquiries can be directed to the corresponding authors.

## Ethics statement

Ethical approval was not required for the study involving humans in accordance with the local legislation and institutional requirements. Written informed consent to participate in this study was not required from the participants or the participants’ legal guardians/next of kin in accordance with the national legislation and the institutional requirements.

## Author contributions

X-yS: Conceptualization, Writing – original draft. Q-kZ: Data curation, Formal analysis, Writing – original draft. JL: Data curation, Formal analysis, Writing – original draft. C-yZ: Data curation, Formal analysis, Writing – original draft. LJ: Writing – review & editing. SF: Writing – review & editing.

## Footnotes

^1^ r9.finngen.fi
