# Supplementary figures and images for: Mendelian randomization analysis reveals causal relationships between circulating cell traits and renal disorders
Source: Front Med (Lausanne). 2024 May 17;11:1360868. doi: 10.3389/fmed.2024.1360868 (PMC11140107; doi:10.3389/fmed.2024.1360868)

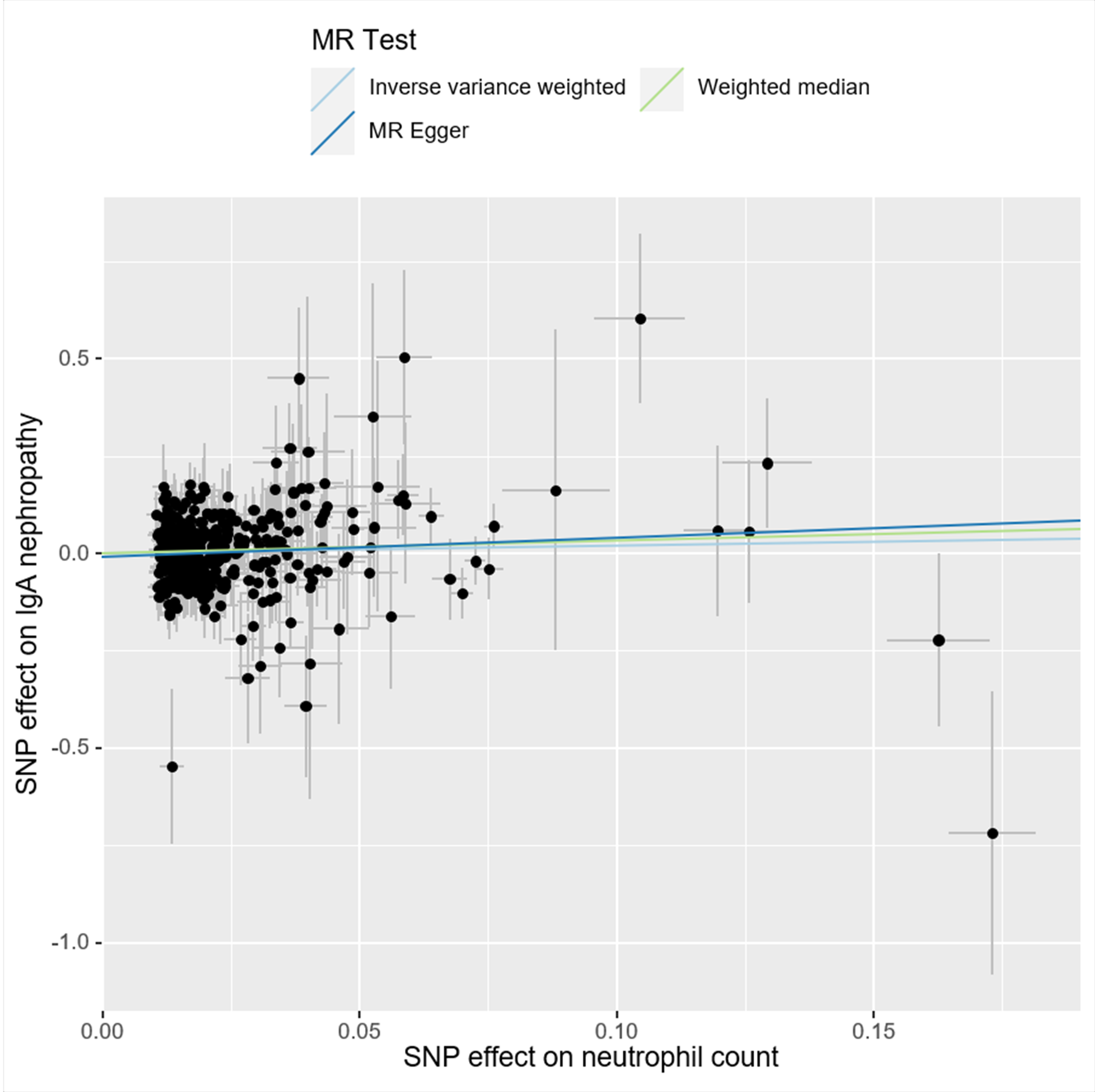

Supplement: Supplementary file 1 [file Data_Sheet_1.ZIP › Supplementary Figure 1.tif]

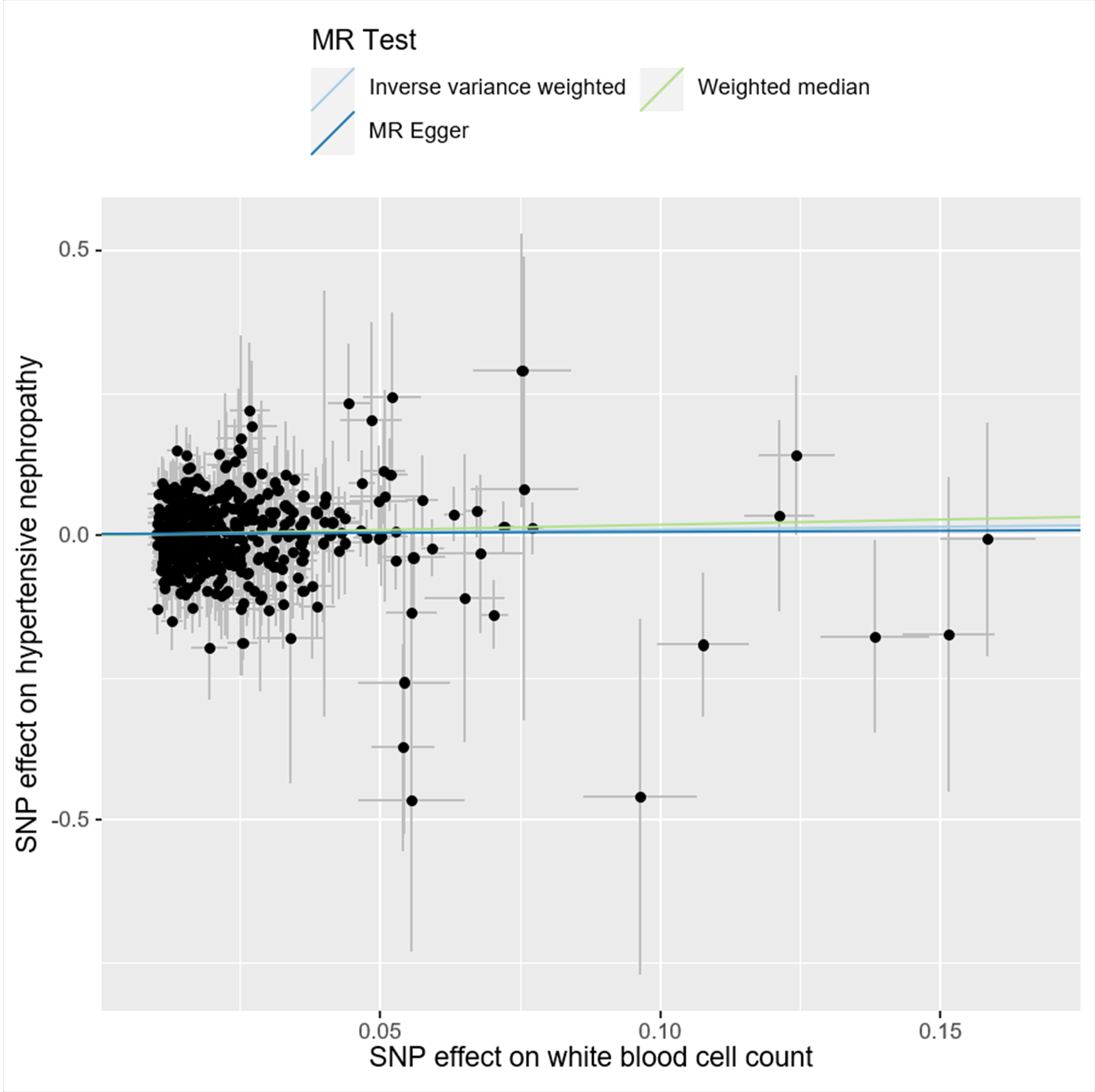

Supplement: Supplementary file 1 [file Data_Sheet_1.ZIP › Supplementary Figure 10.tif]

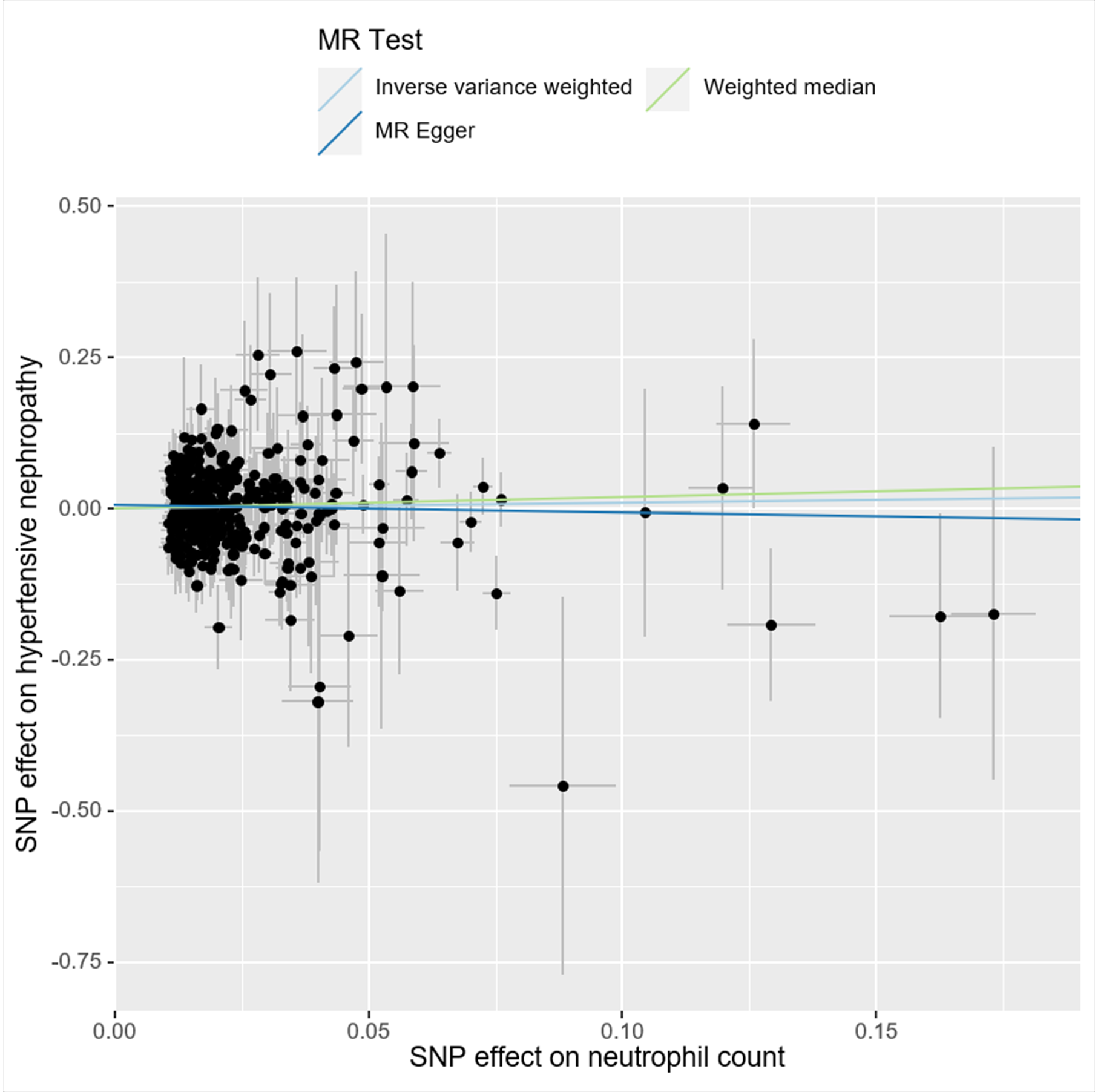

Supplement: Supplementary file 1 [file Data_Sheet_1.ZIP › Supplementary Figure 11.tif]

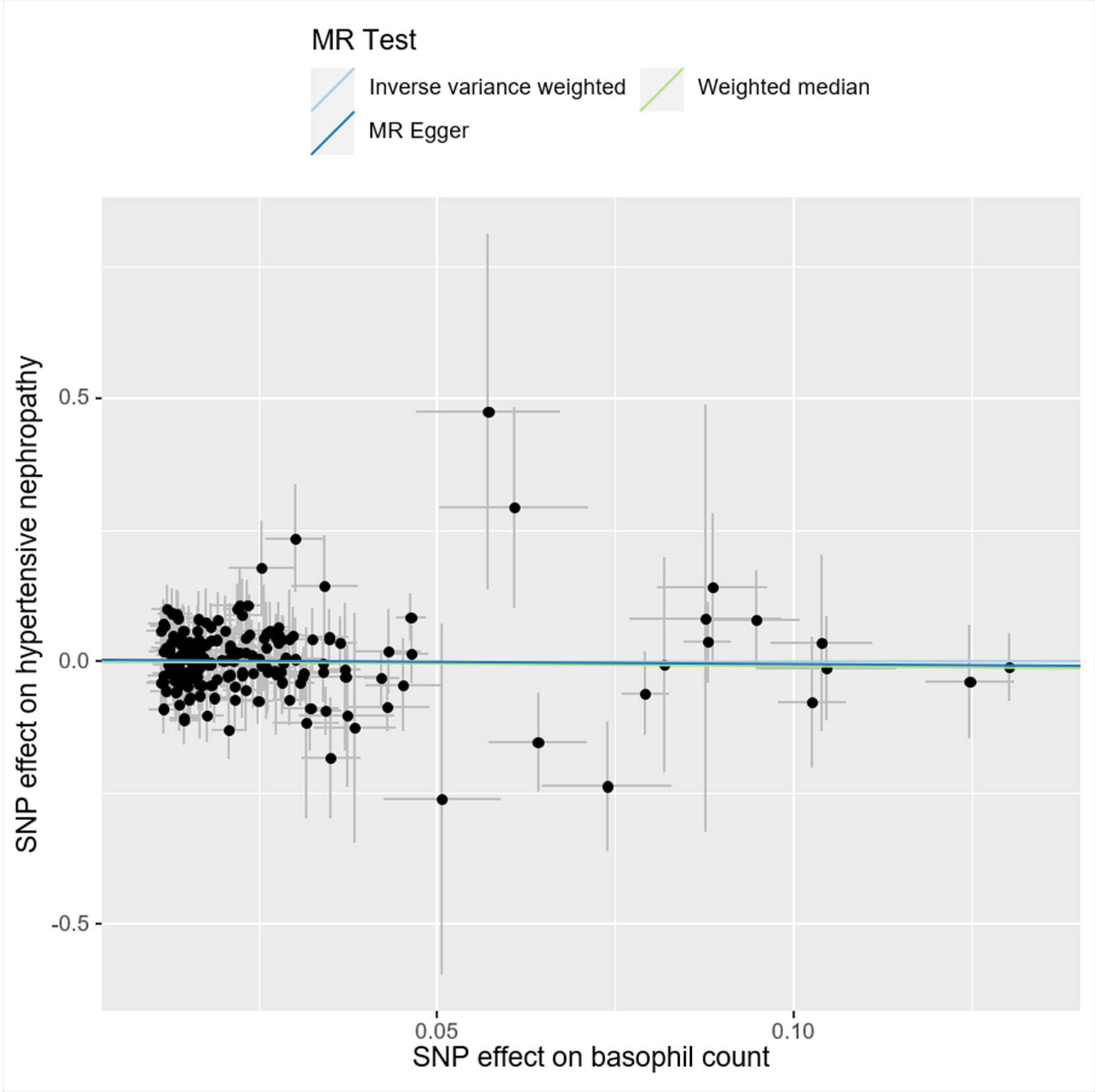

Supplement: Supplementary file 1 [file Data_Sheet_1.ZIP › Supplementary Figure 12.tif]

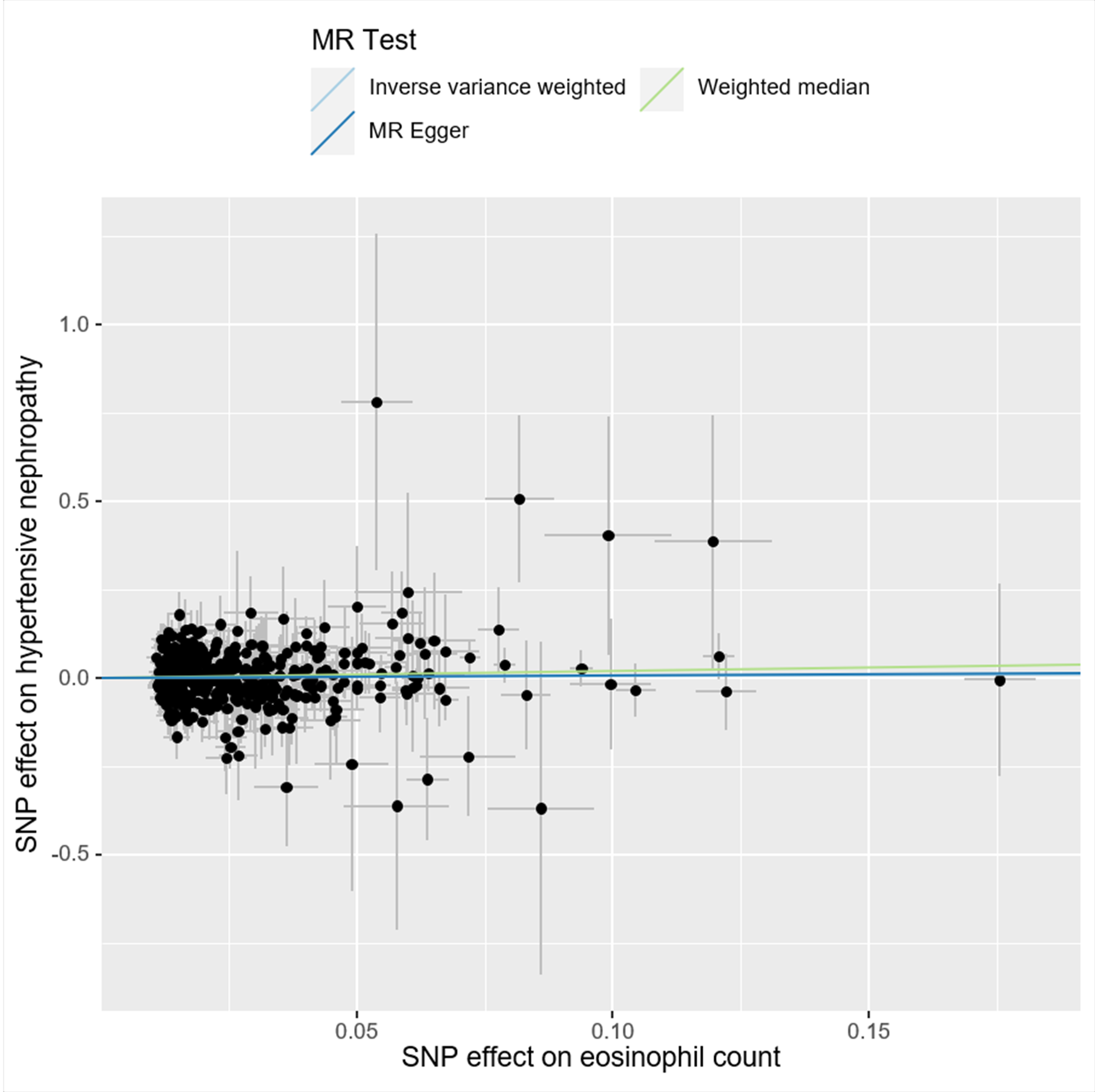

Supplement: Supplementary file 1 [file Data_Sheet_1.ZIP › Supplementary Figure 13.tif]

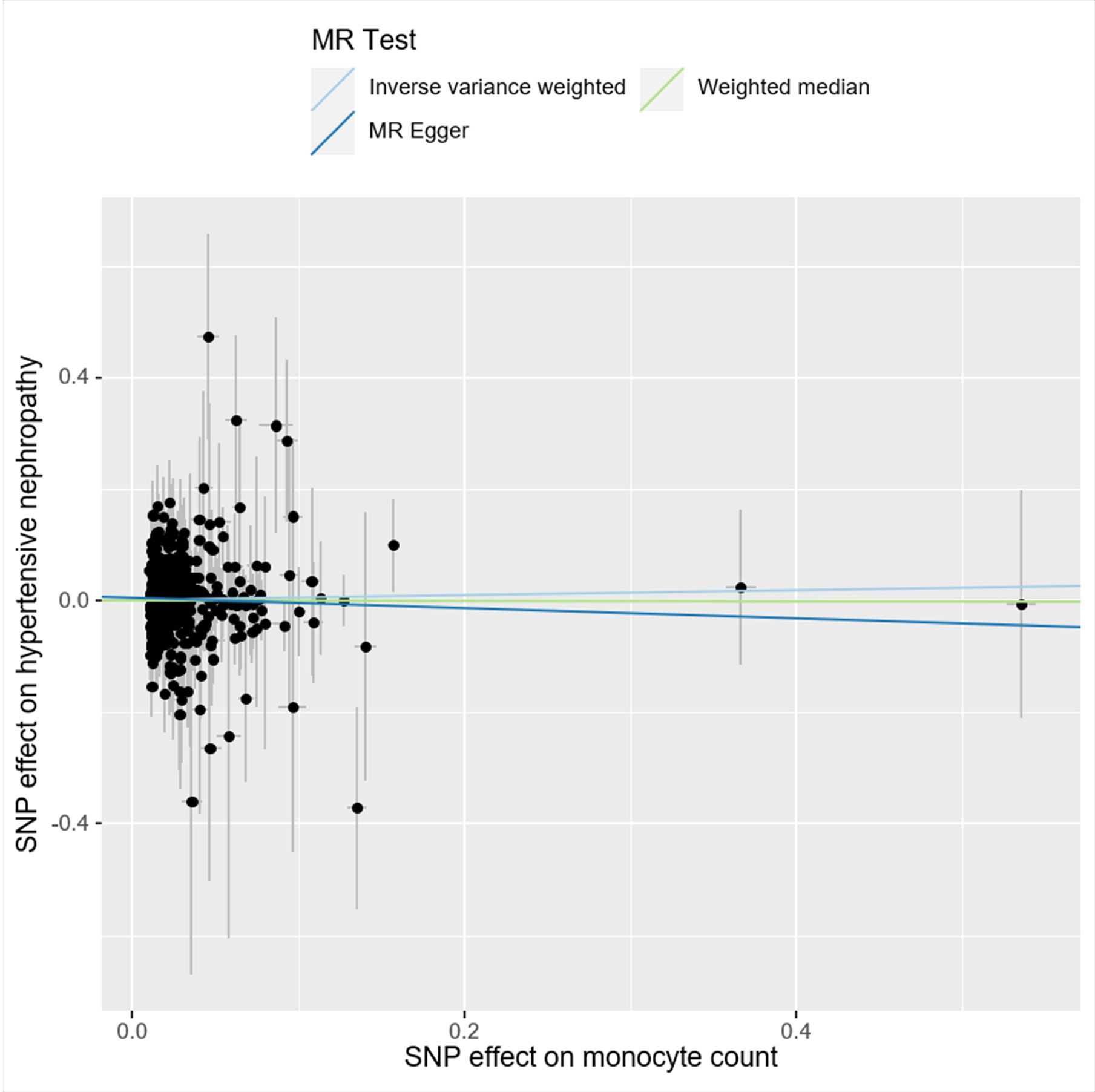

Supplement: Supplementary file 1 [file Data_Sheet_1.ZIP › Supplementary Figure 14.tif]

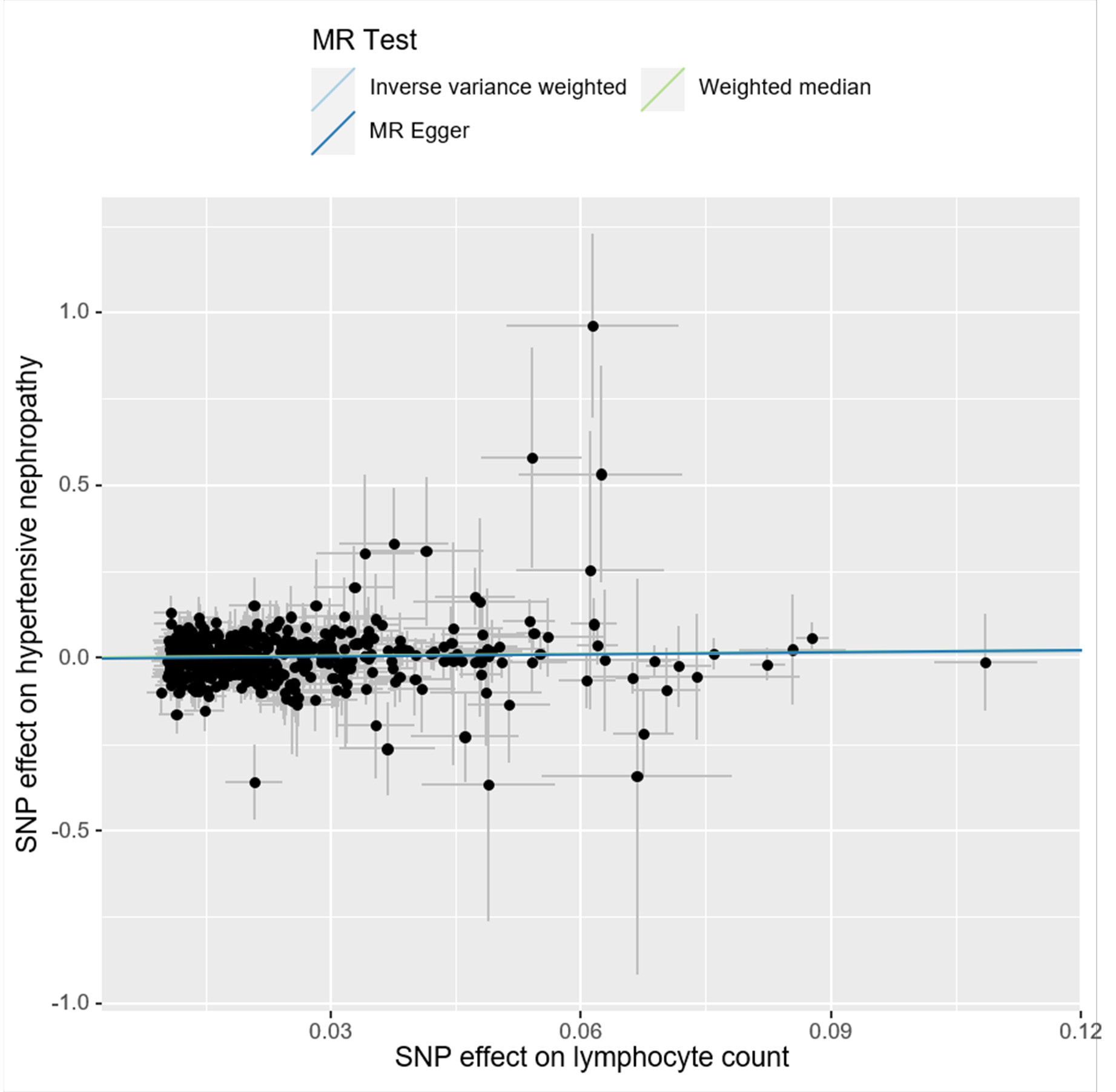

Supplement: Supplementary file 1 [file Data_Sheet_1.ZIP › Supplementary Figure 15.tif]

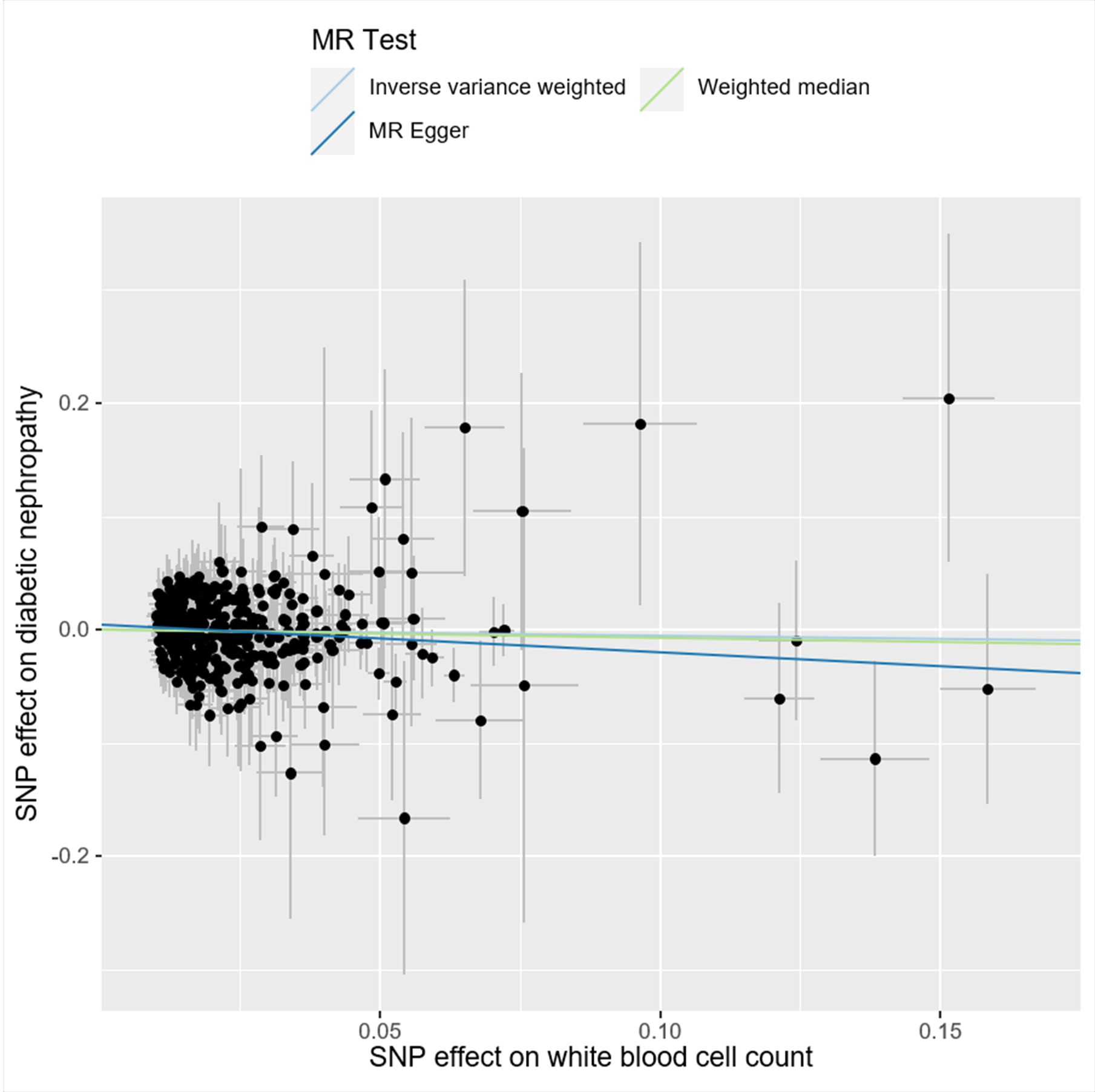

Supplement: Supplementary file 1 [file Data_Sheet_1.ZIP › Supplementary Figure 16.tif]

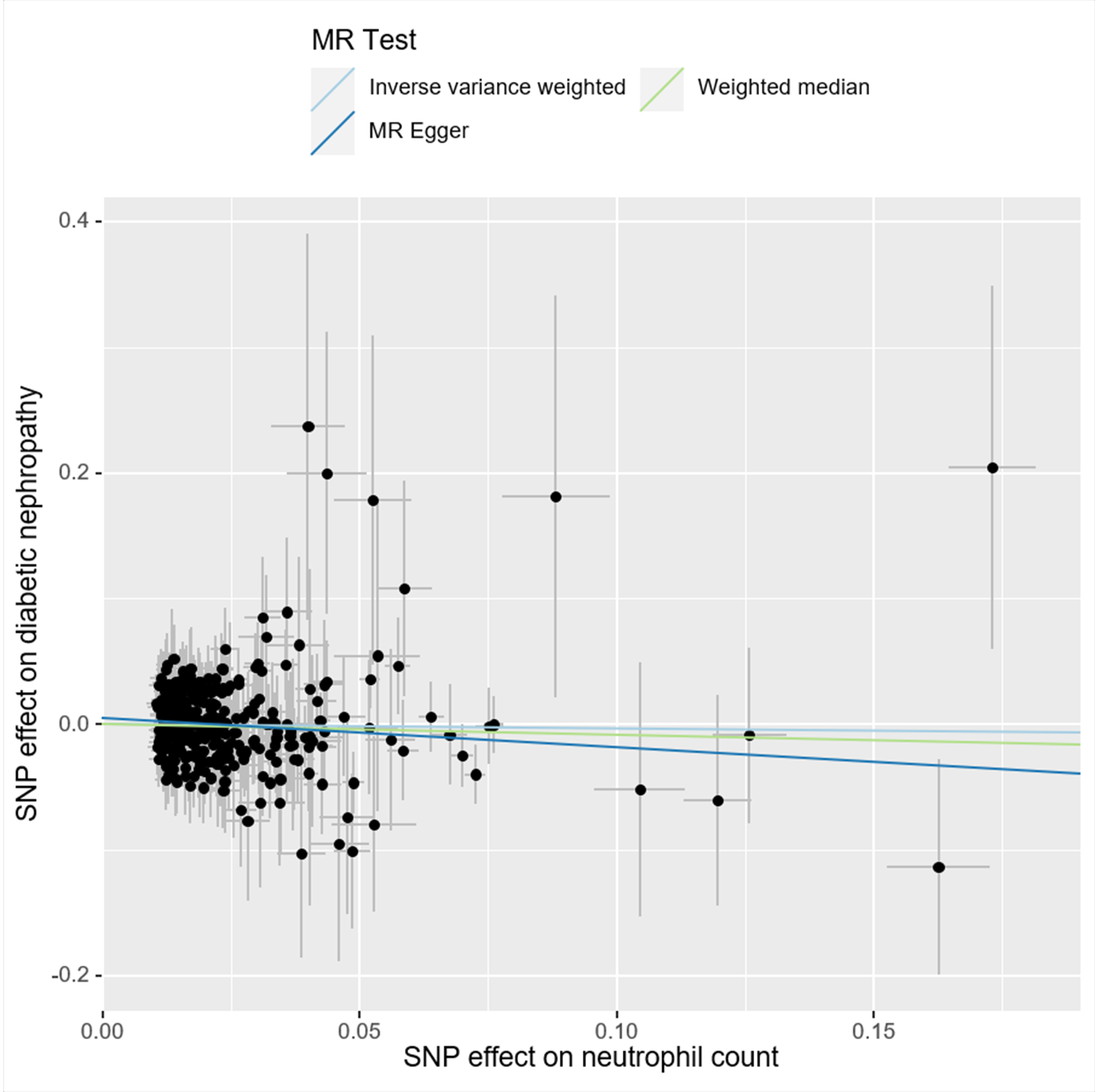

Supplement: Supplementary file 1 [file Data_Sheet_1.ZIP › Supplementary Figure 17.tif]

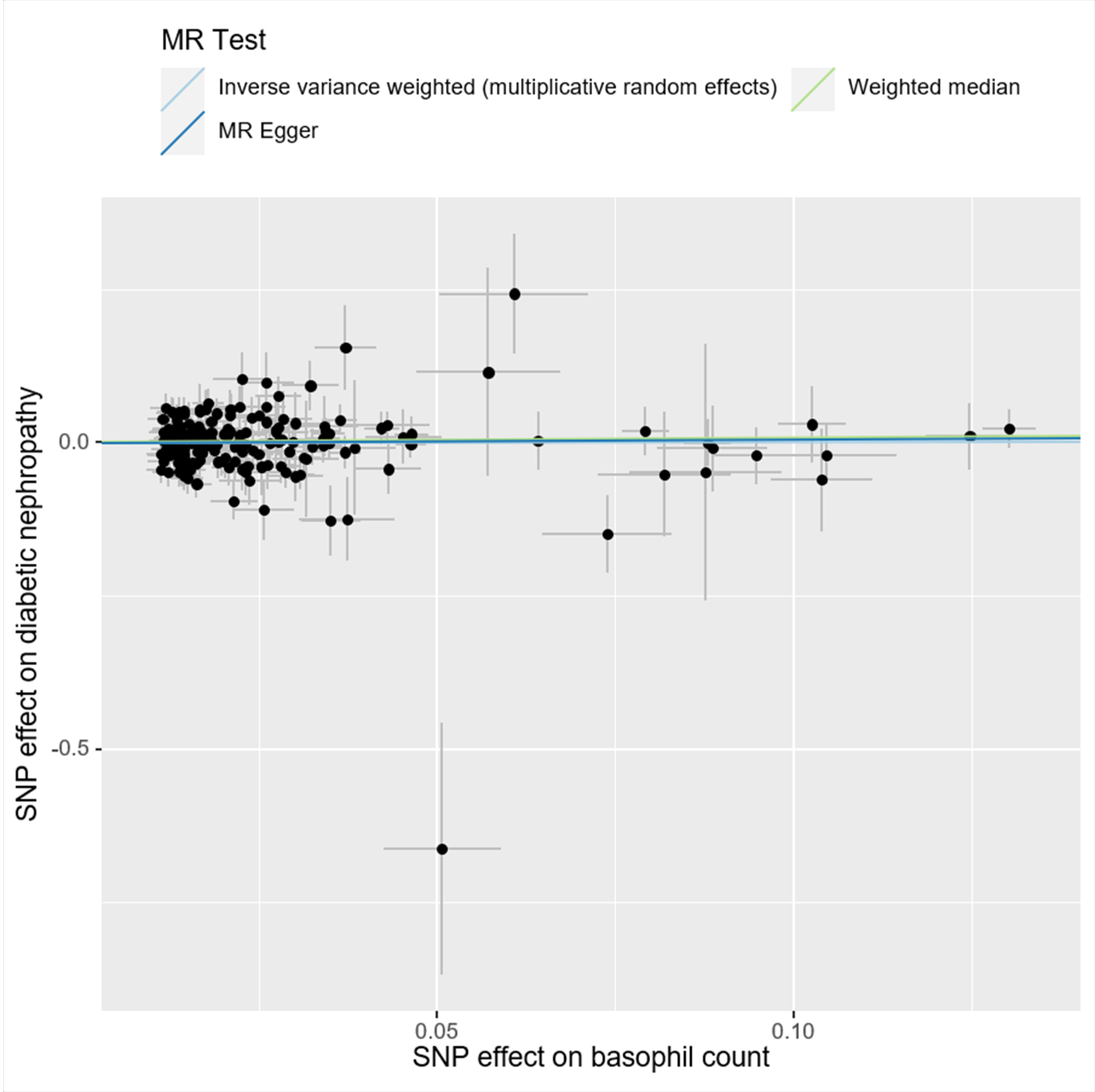

Supplement: Supplementary file 1 [file Data_Sheet_1.ZIP › Supplementary Figure 18.tif]

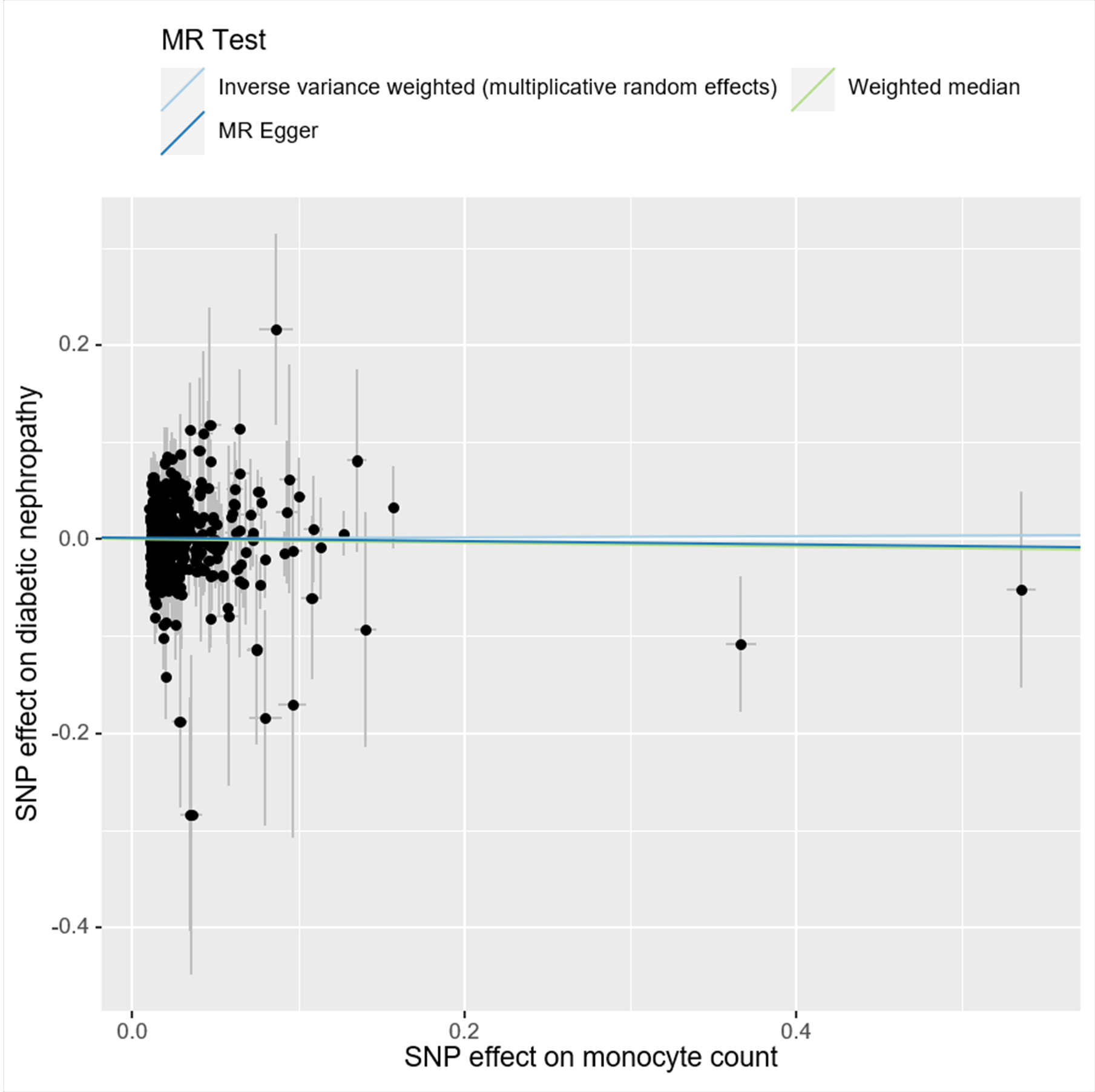

Supplement: Supplementary file 1 [file Data_Sheet_1.ZIP › Supplementary Figure 19.tif]

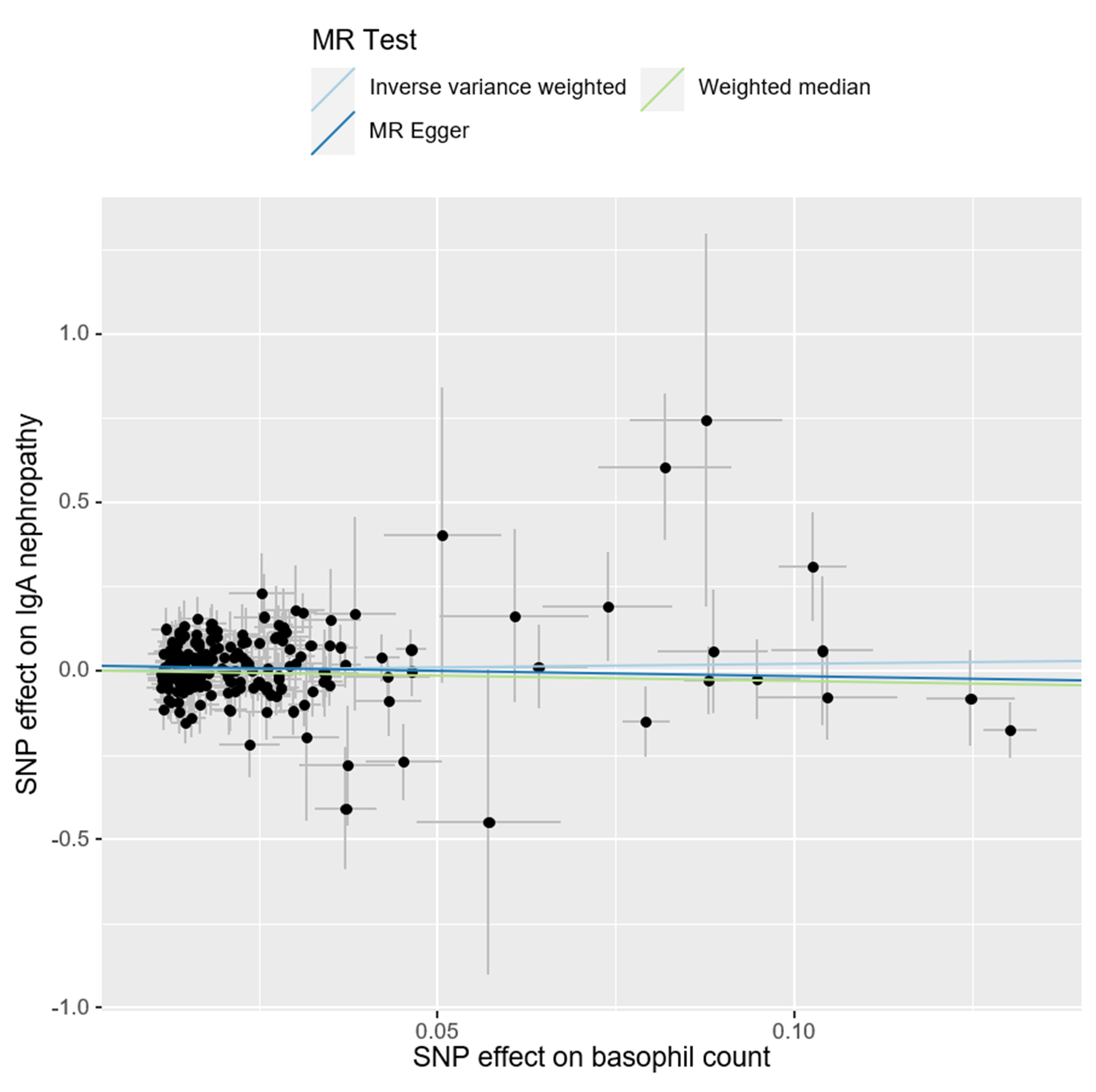

Supplement: Supplementary file 1 [file Data_Sheet_1.ZIP › Supplementary Figure 2.tif]

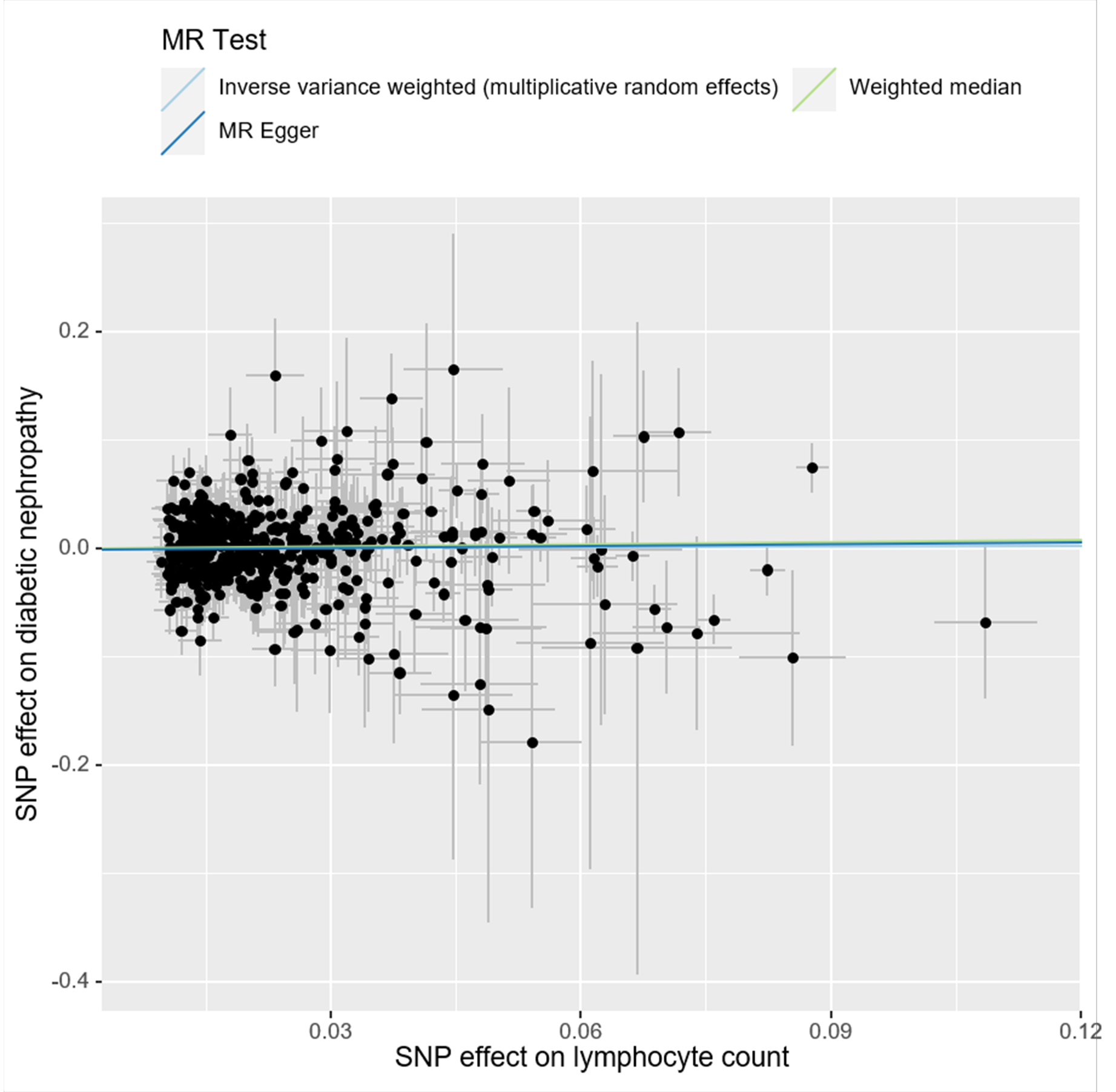

Supplement: Supplementary file 1 [file Data_Sheet_1.ZIP › Supplementary Figure 20.tif]

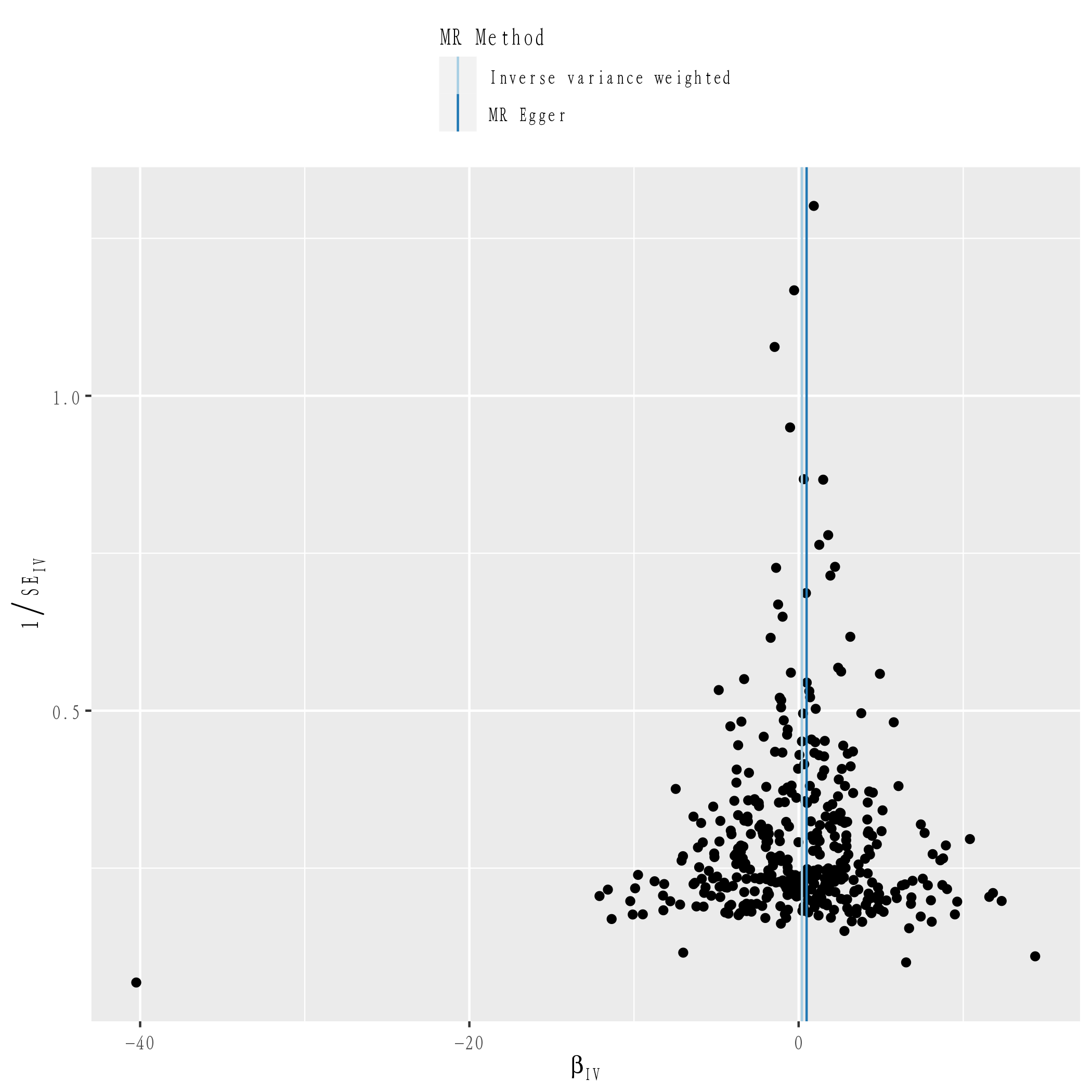

Supplement: Supplementary file 1 [file Data_Sheet_1.ZIP › Supplementary Figure 21.tif]

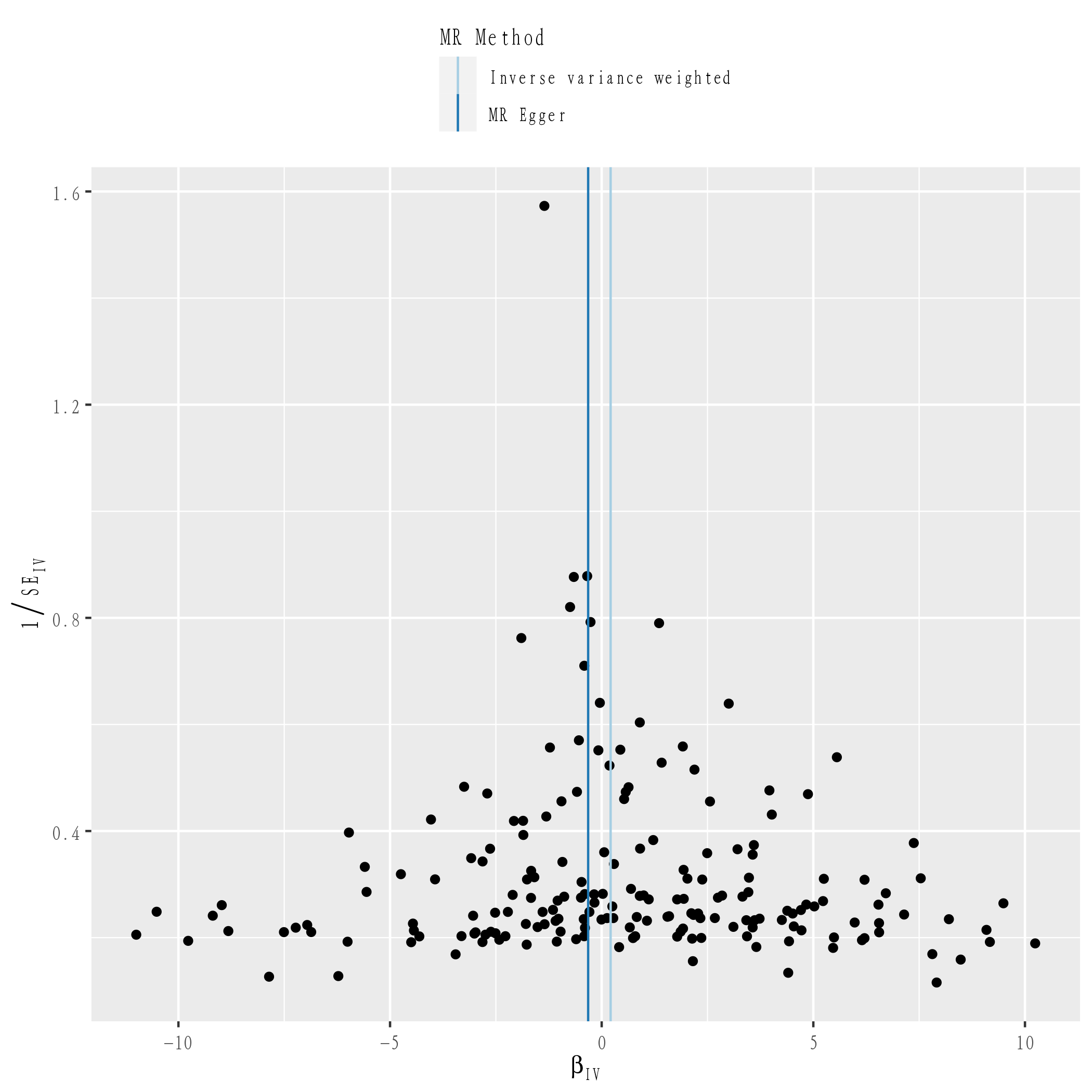

Supplement: Supplementary file 1 [file Data_Sheet_1.ZIP › Supplementary Figure 22.tif]

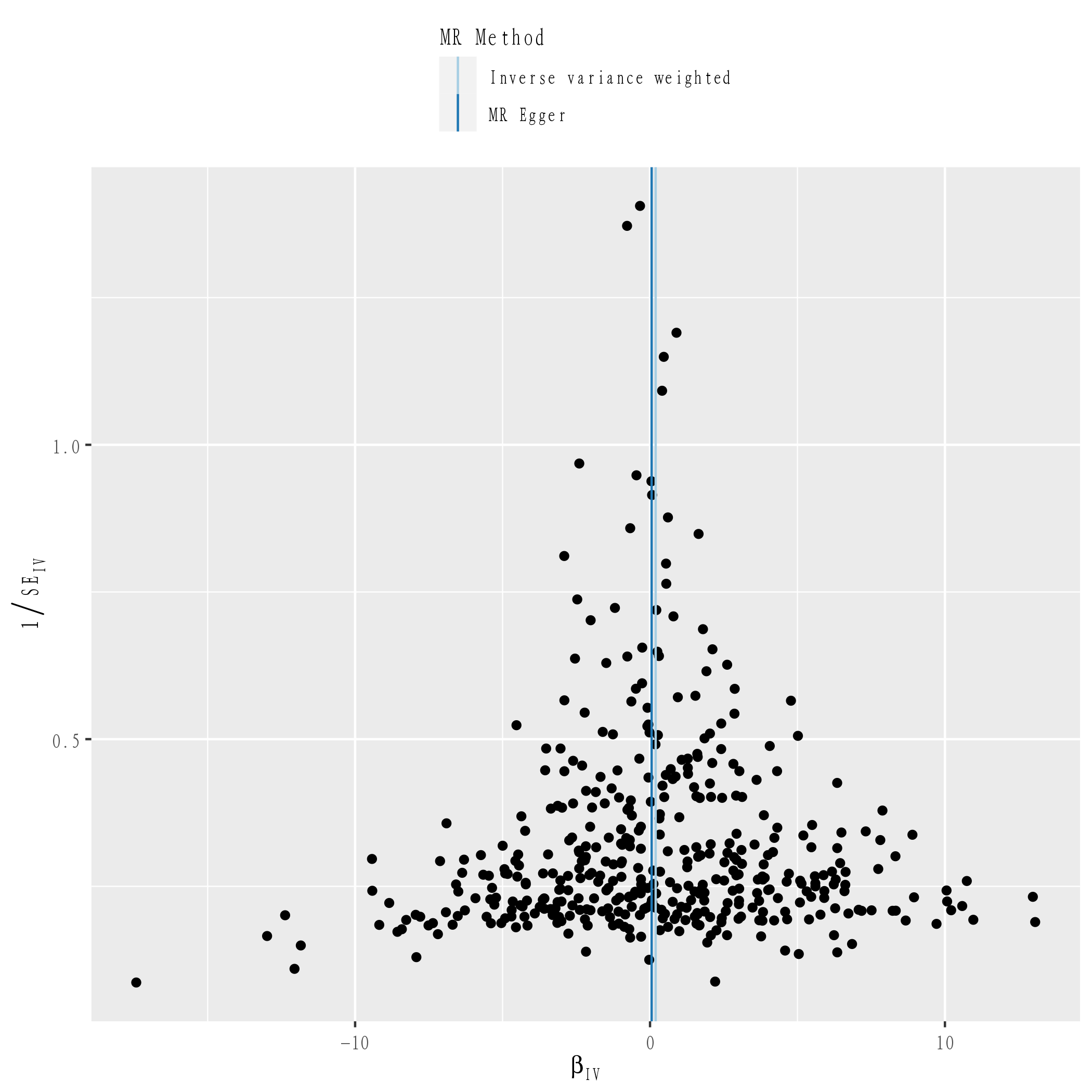

Supplement: Supplementary file 1 [file Data_Sheet_1.ZIP › Supplementary Figure 23.tif]

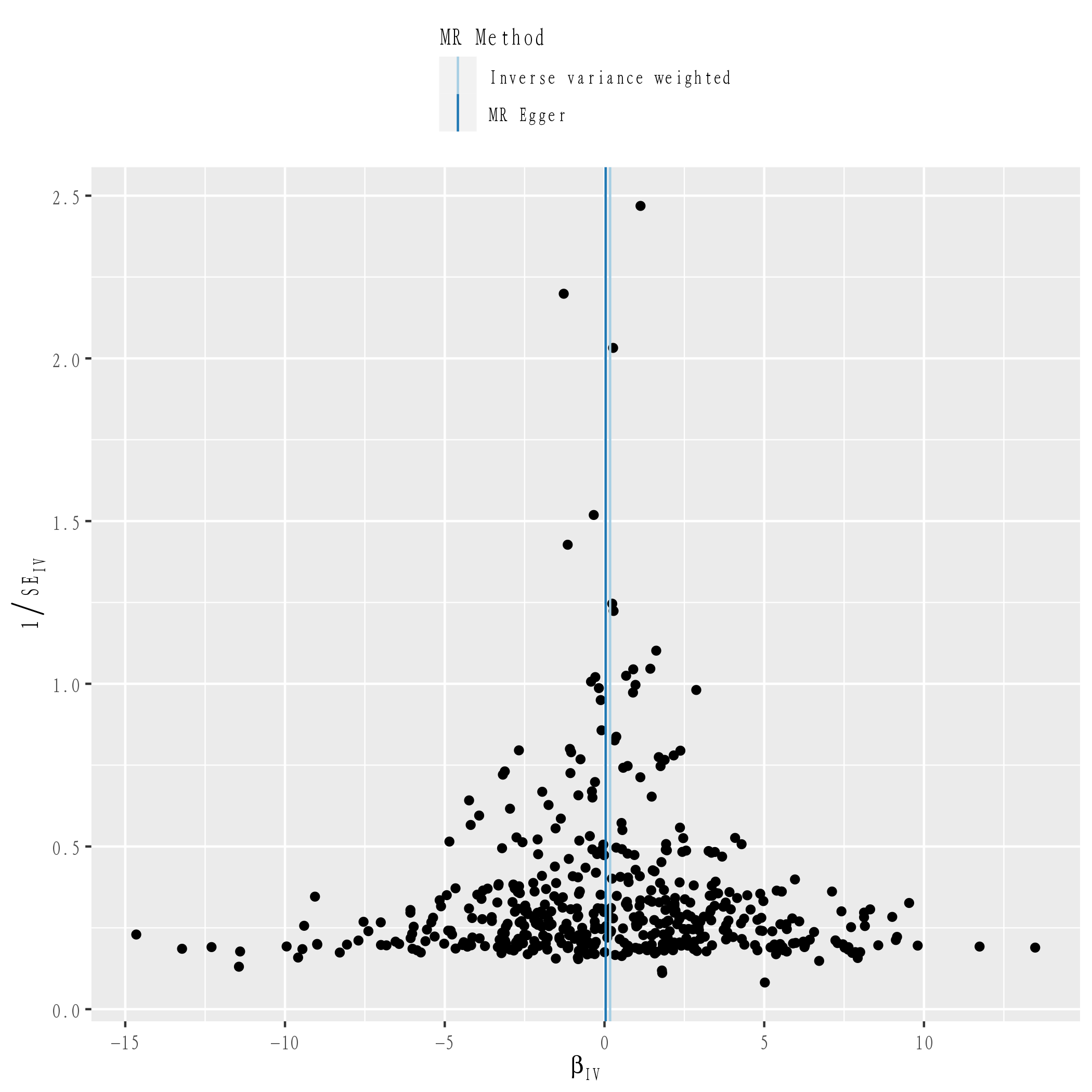

Supplement: Supplementary file 1 [file Data_Sheet_1.ZIP › Supplementary Figure 24.tif]

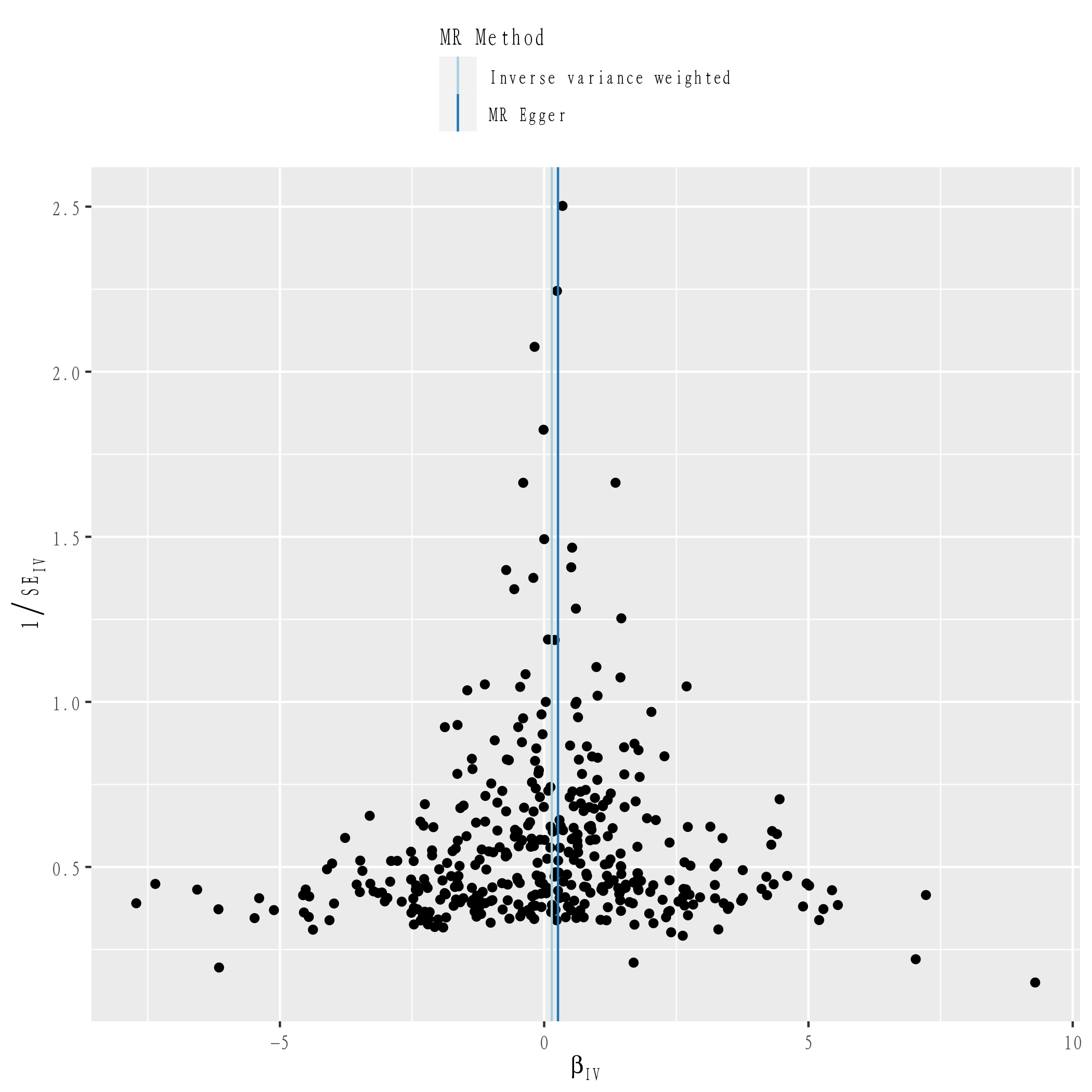

Supplement: Supplementary file 1 [file Data_Sheet_1.ZIP › Supplementary Figure 25.tif]

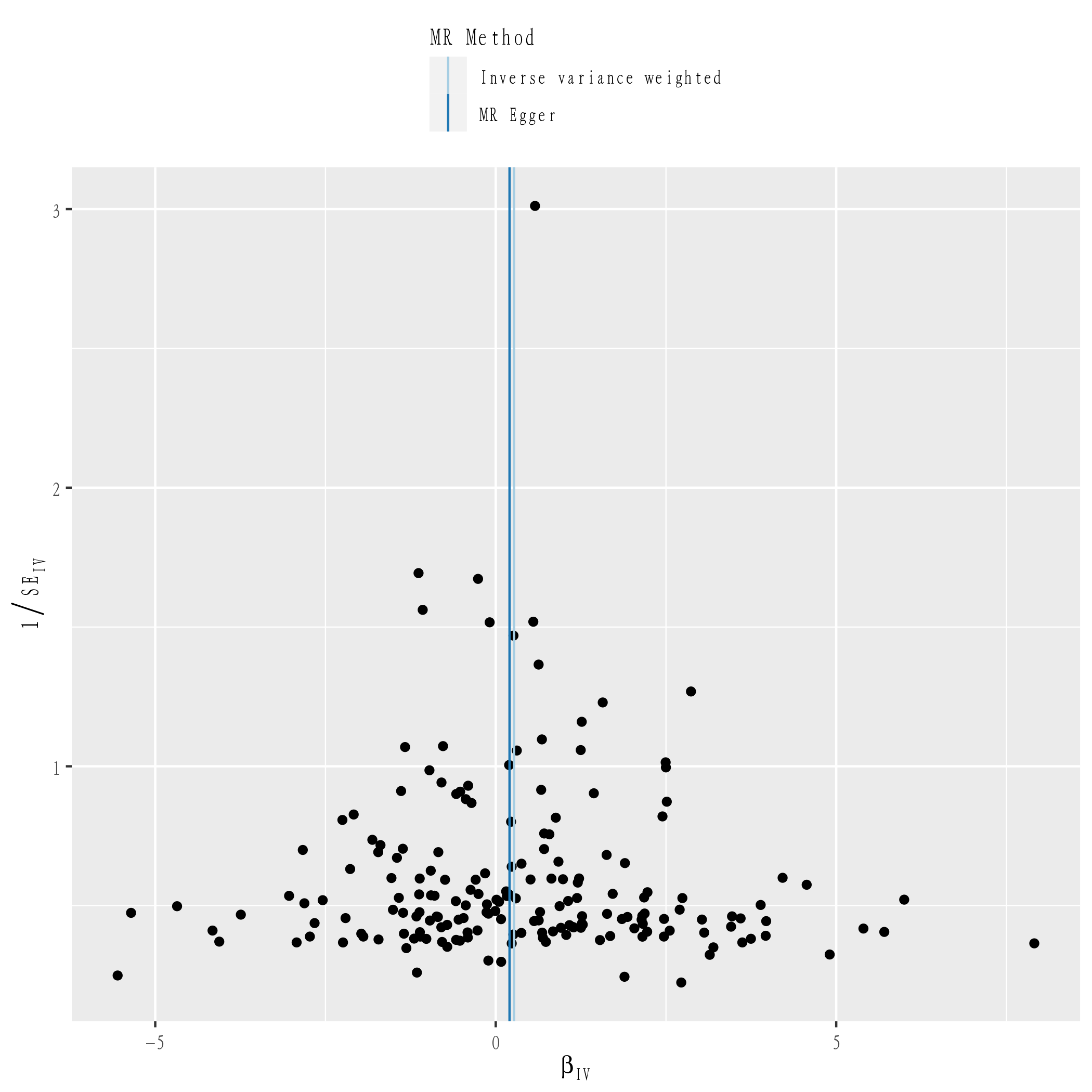

Supplement: Supplementary file 1 [file Data_Sheet_1.ZIP › Supplementary Figure 26.tif]

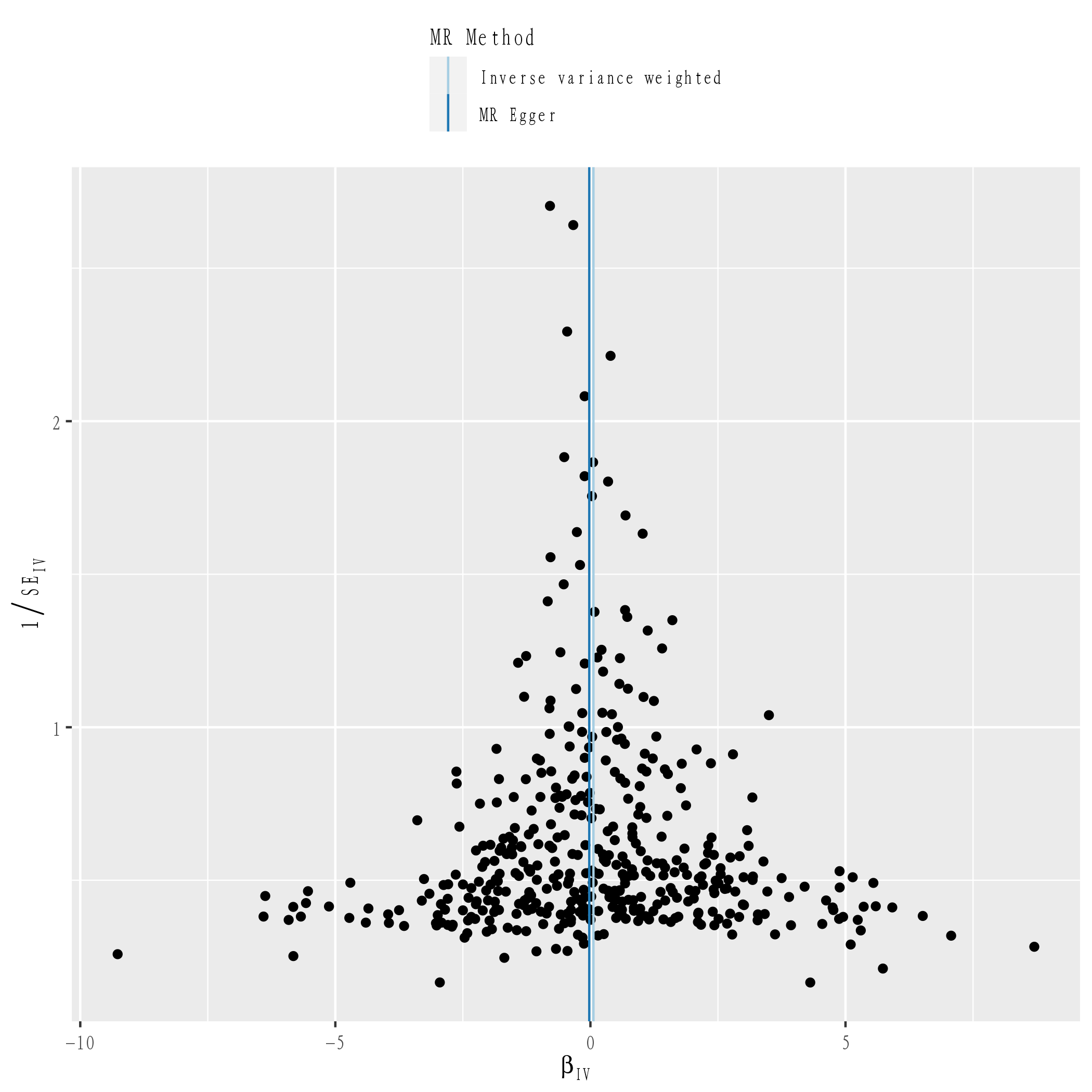

Supplement: Supplementary file 1 [file Data_Sheet_1.ZIP › Supplementary Figure 27.tif]

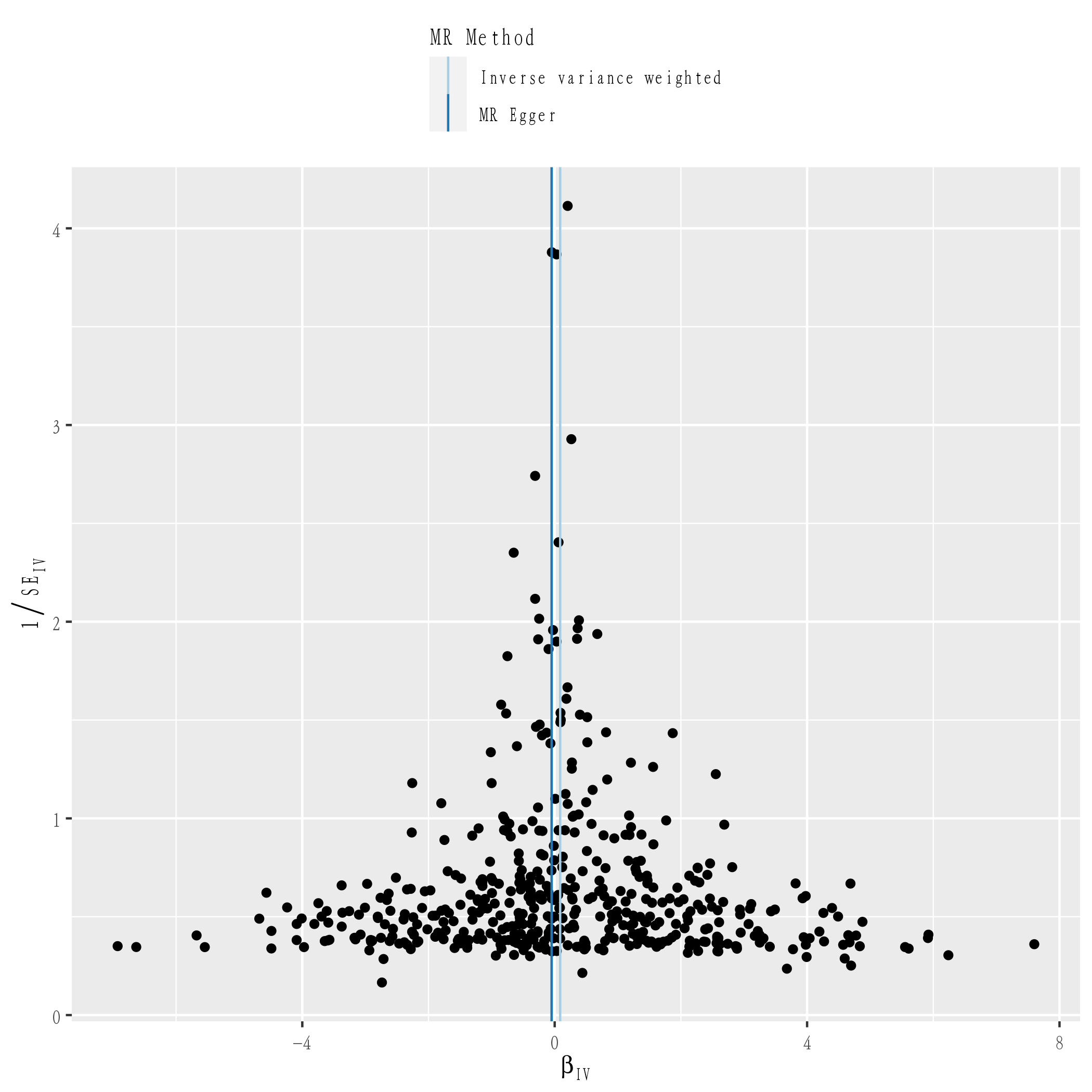

Supplement: Supplementary file 1 [file Data_Sheet_1.ZIP › Supplementary Figure 28.tif]

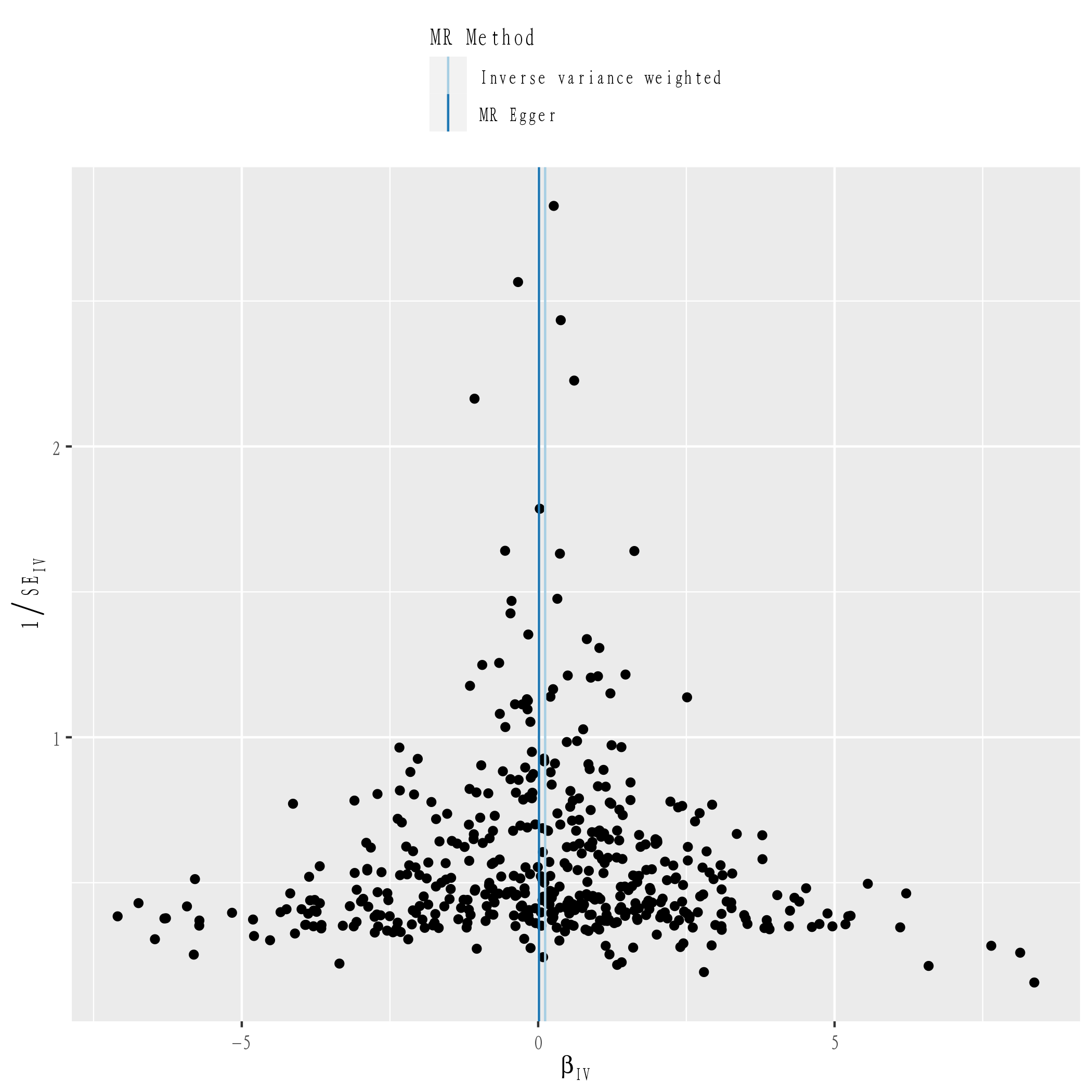

Supplement: Supplementary file 1 [file Data_Sheet_1.ZIP › Supplementary Figure 29.tif]

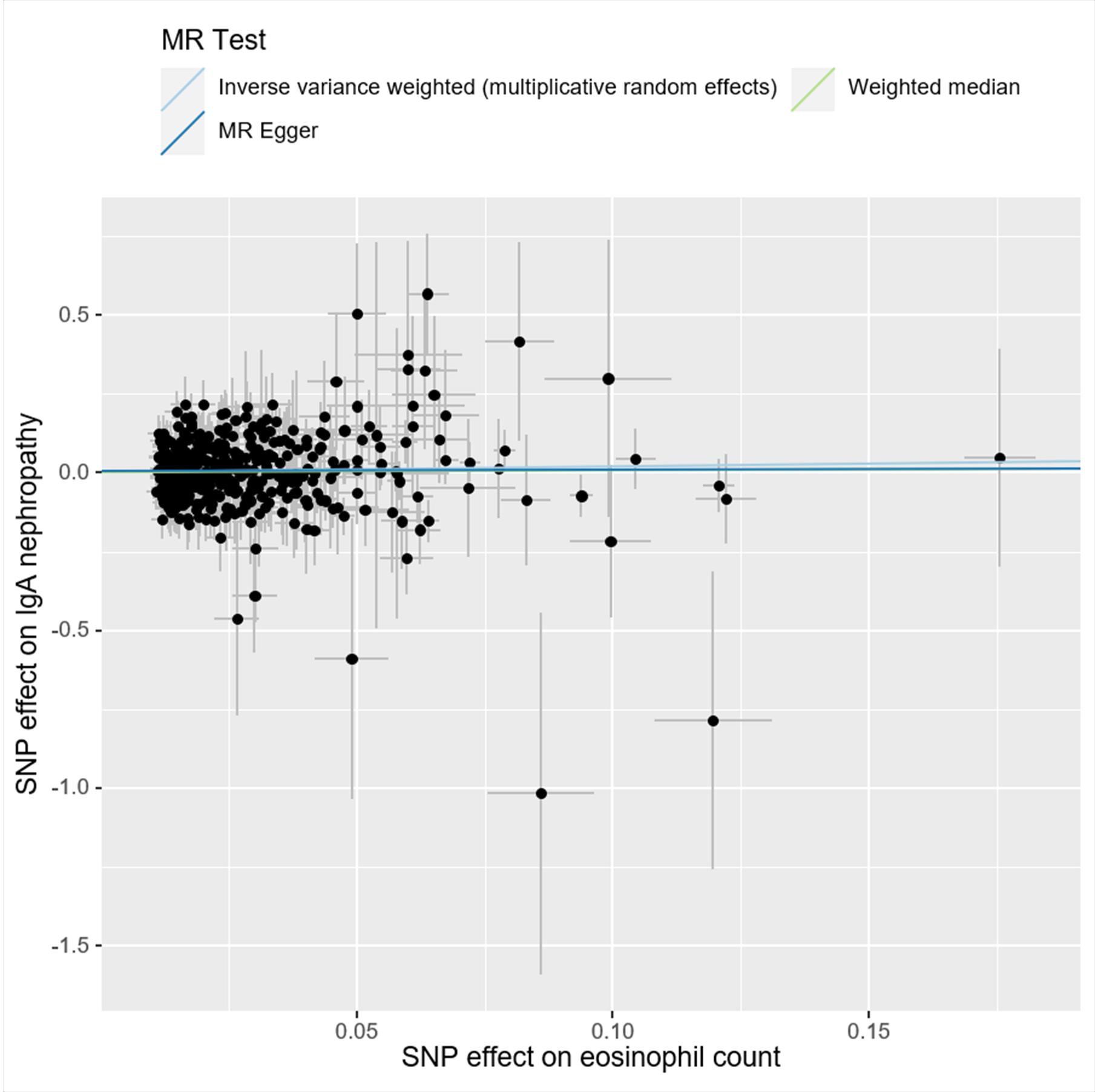

Supplement: Supplementary file 1 [file Data_Sheet_1.ZIP › Supplementary Figure 3.tif]

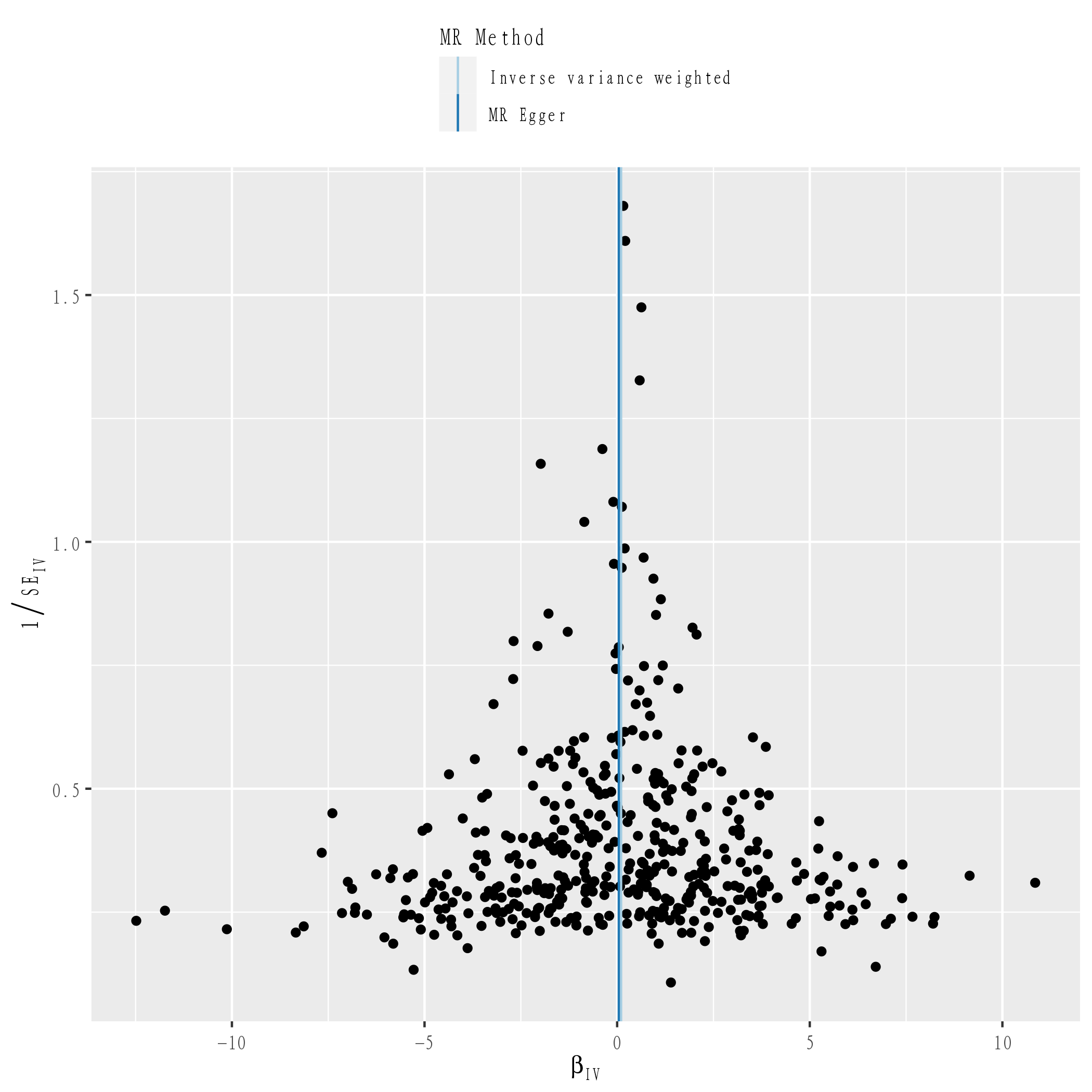

Supplement: Supplementary file 1 [file Data_Sheet_1.ZIP › Supplementary Figure 30.tif]

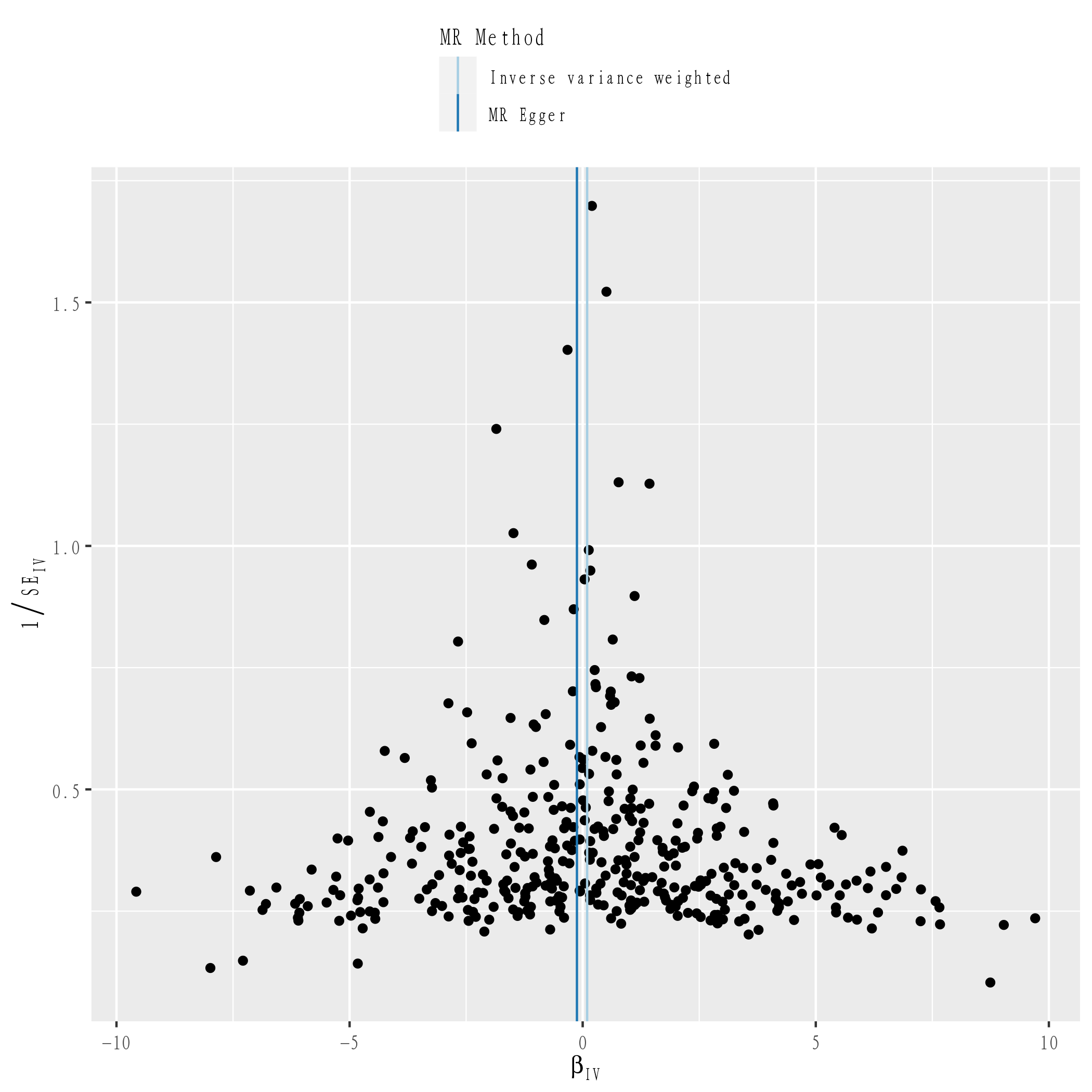

Supplement: Supplementary file 1 [file Data_Sheet_1.ZIP › Supplementary Figure 31.tif]

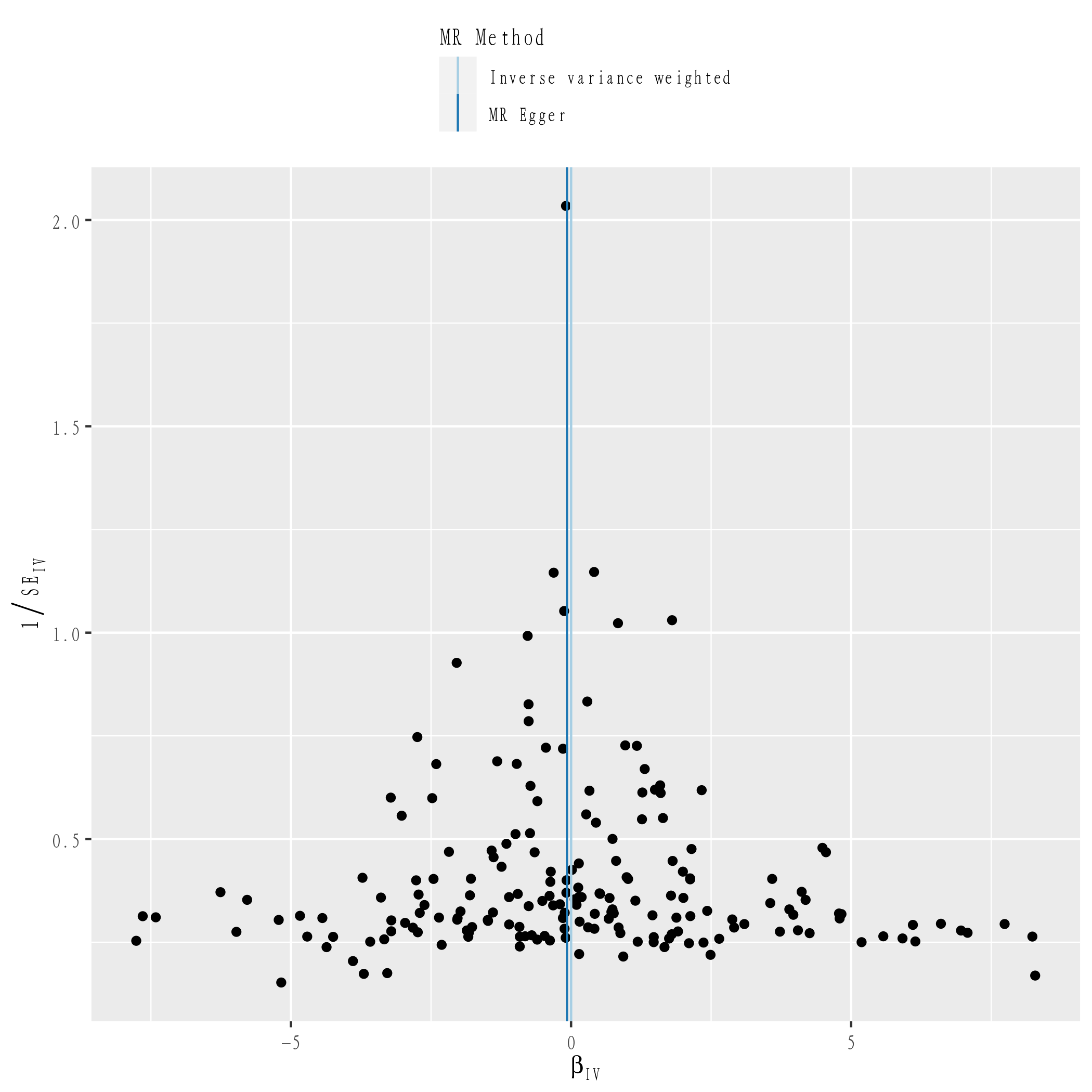

Supplement: Supplementary file 1 [file Data_Sheet_1.ZIP › Supplementary Figure 32.tif]

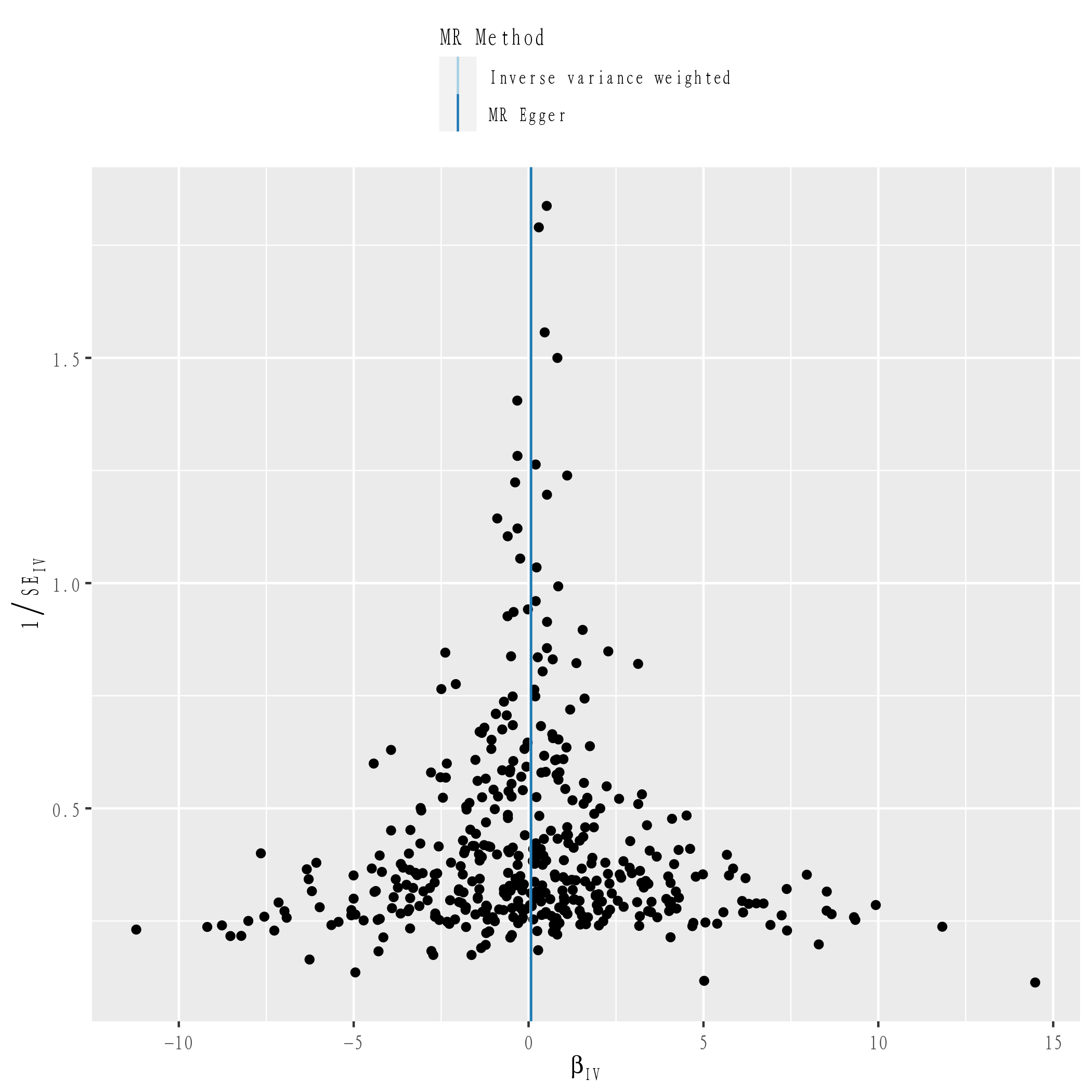

Supplement: Supplementary file 1 [file Data_Sheet_1.ZIP › Supplementary Figure 33.tif]

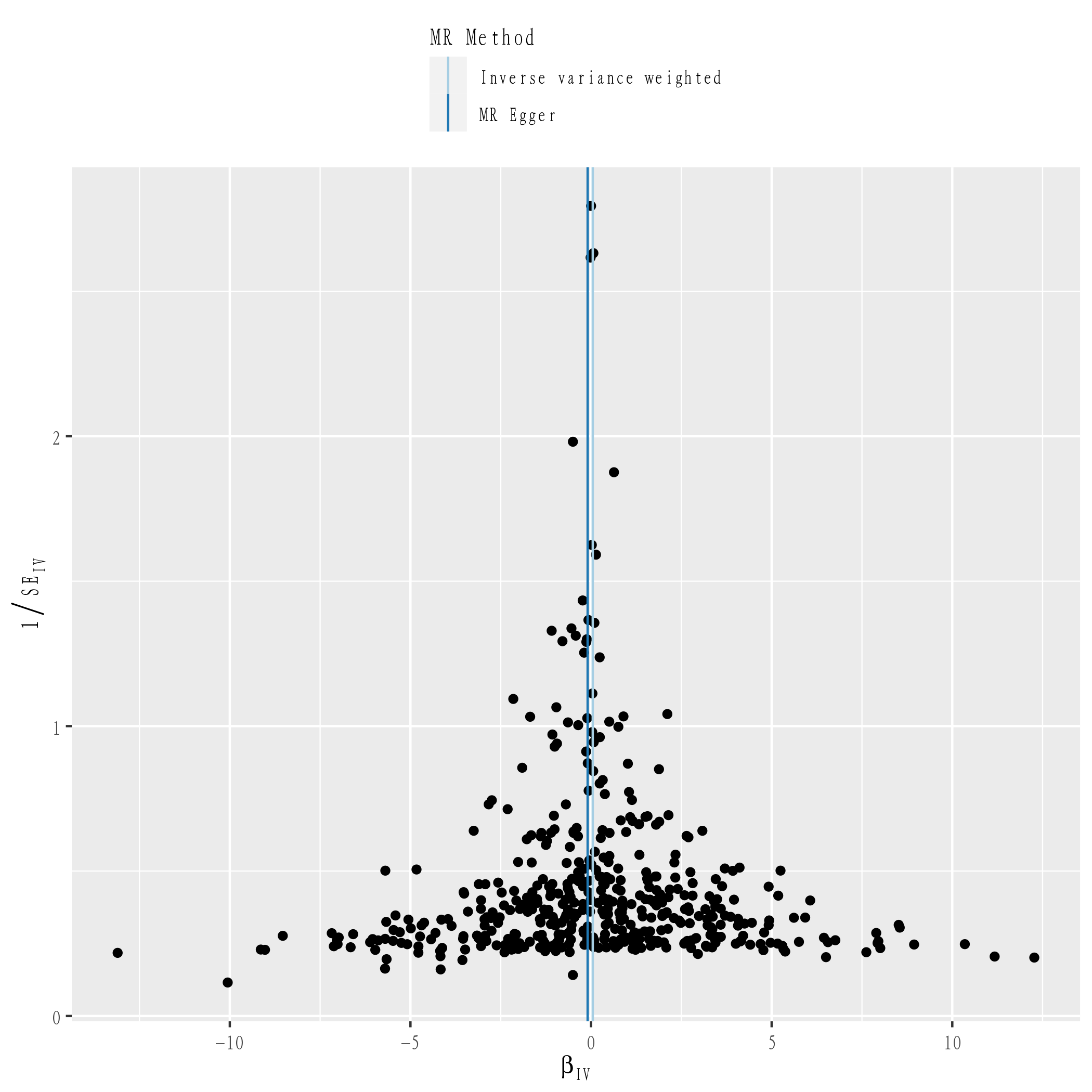

Supplement: Supplementary file 1 [file Data_Sheet_1.ZIP › Supplementary Figure 34.tif]

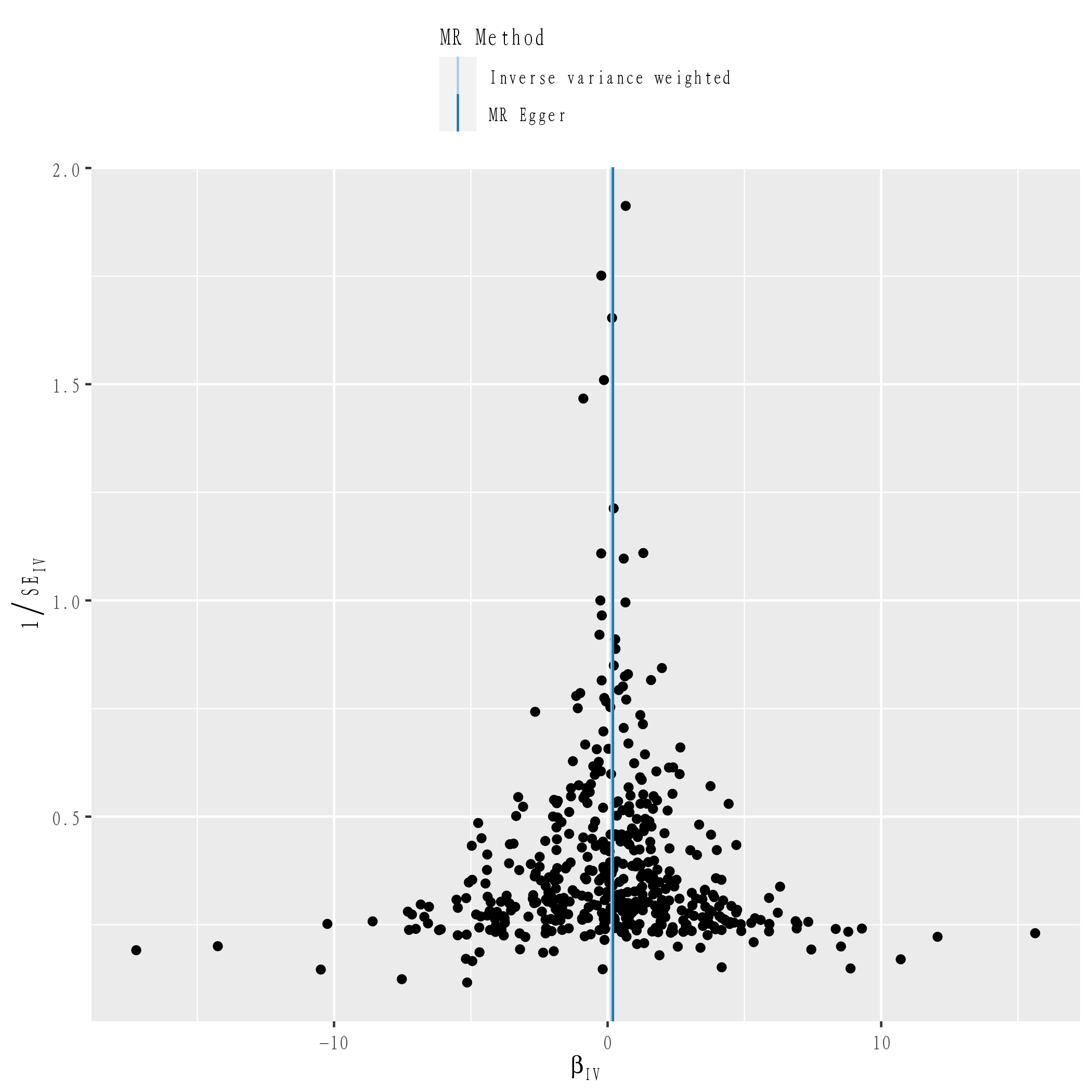

Supplement: Supplementary file 1 [file Data_Sheet_1.ZIP › Supplementary Figure 35.tif]

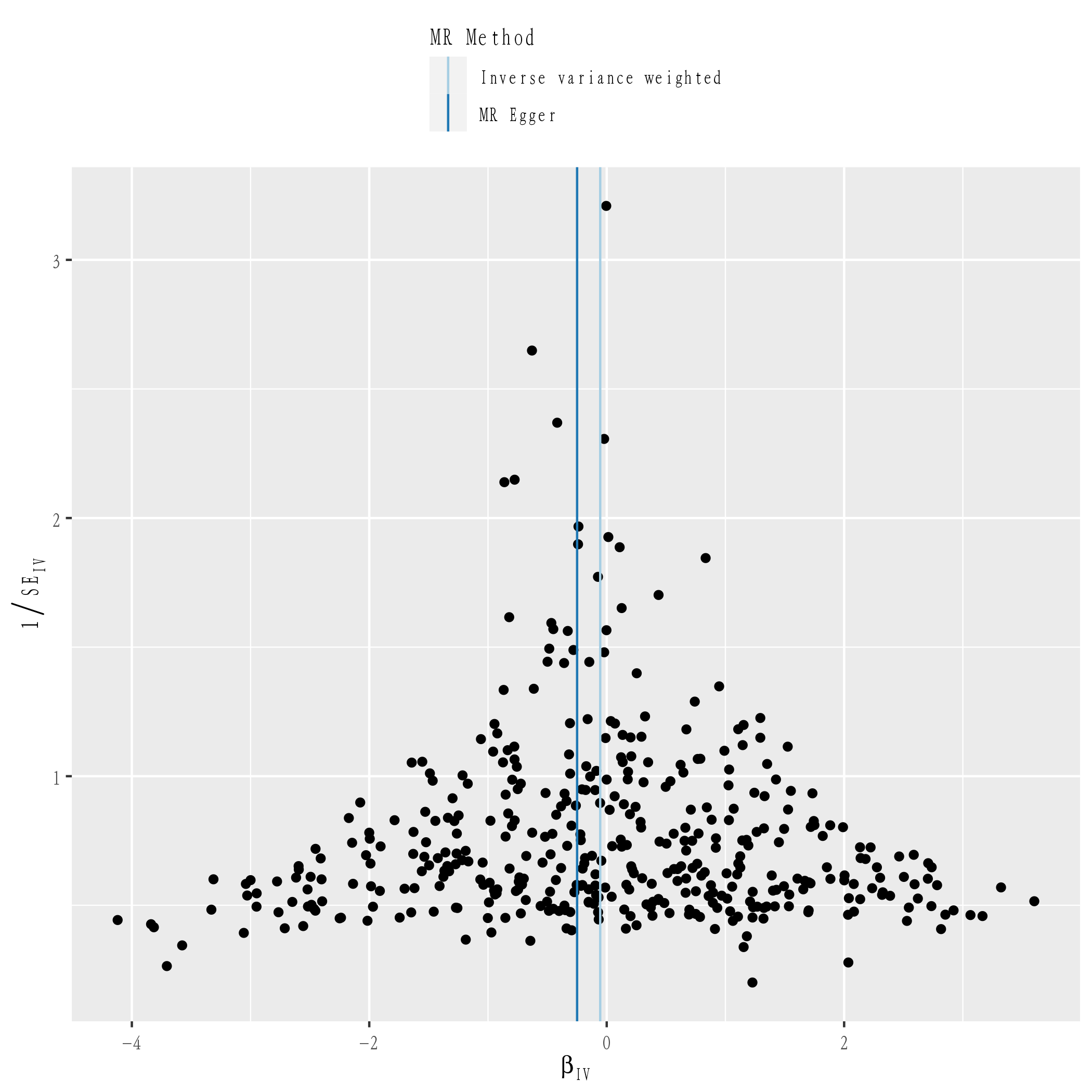

Supplement: Supplementary file 1 [file Data_Sheet_1.ZIP › Supplementary Figure 36.tif]

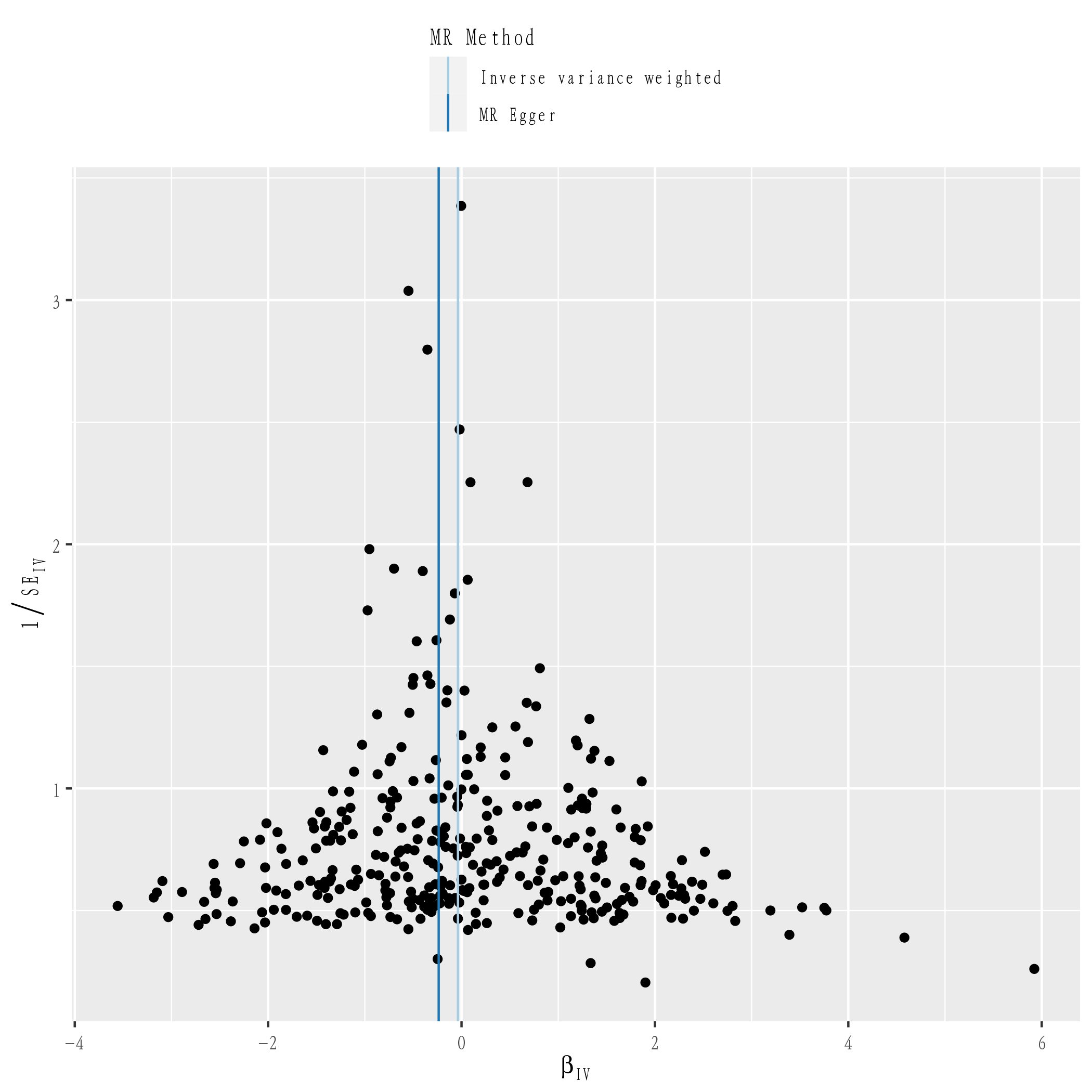

Supplement: Supplementary file 1 [file Data_Sheet_1.ZIP › Supplementary Figure 37.tif]

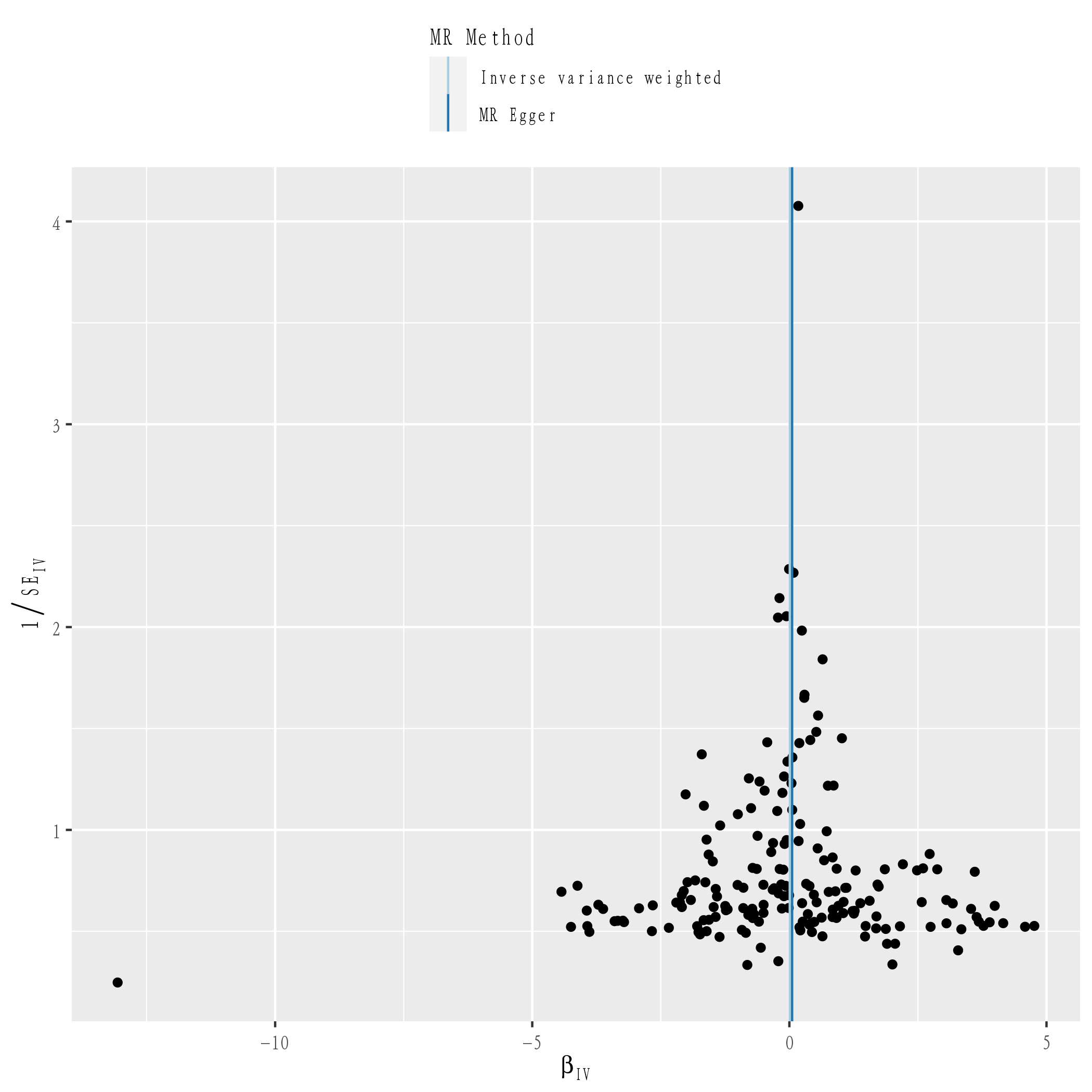

Supplement: Supplementary file 1 [file Data_Sheet_1.ZIP › Supplementary Figure 38.tif]

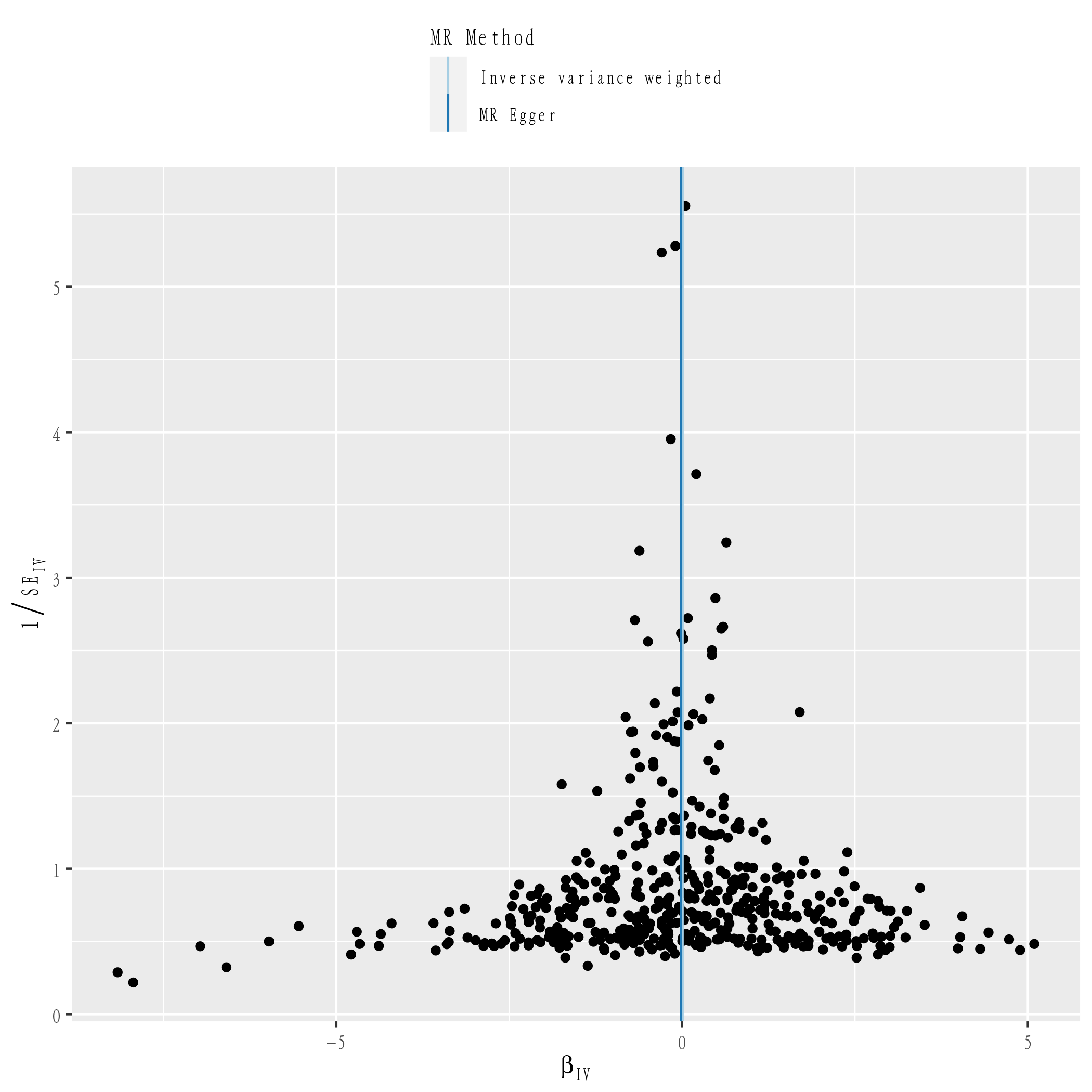

Supplement: Supplementary file 1 [file Data_Sheet_1.ZIP › Supplementary Figure 39.tif]

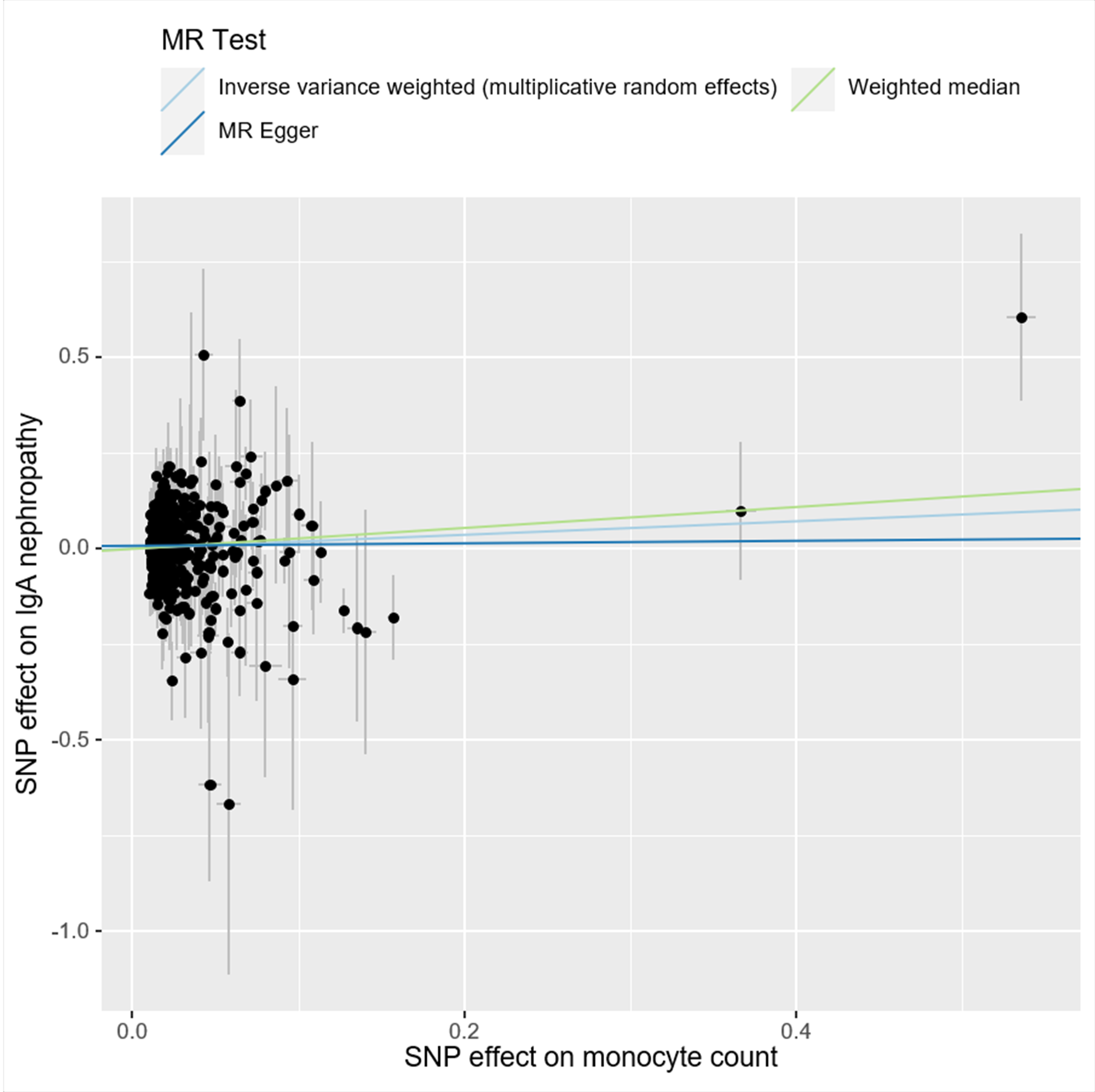

Supplement: Supplementary file 1 [file Data_Sheet_1.ZIP › Supplementary Figure 4.tif]

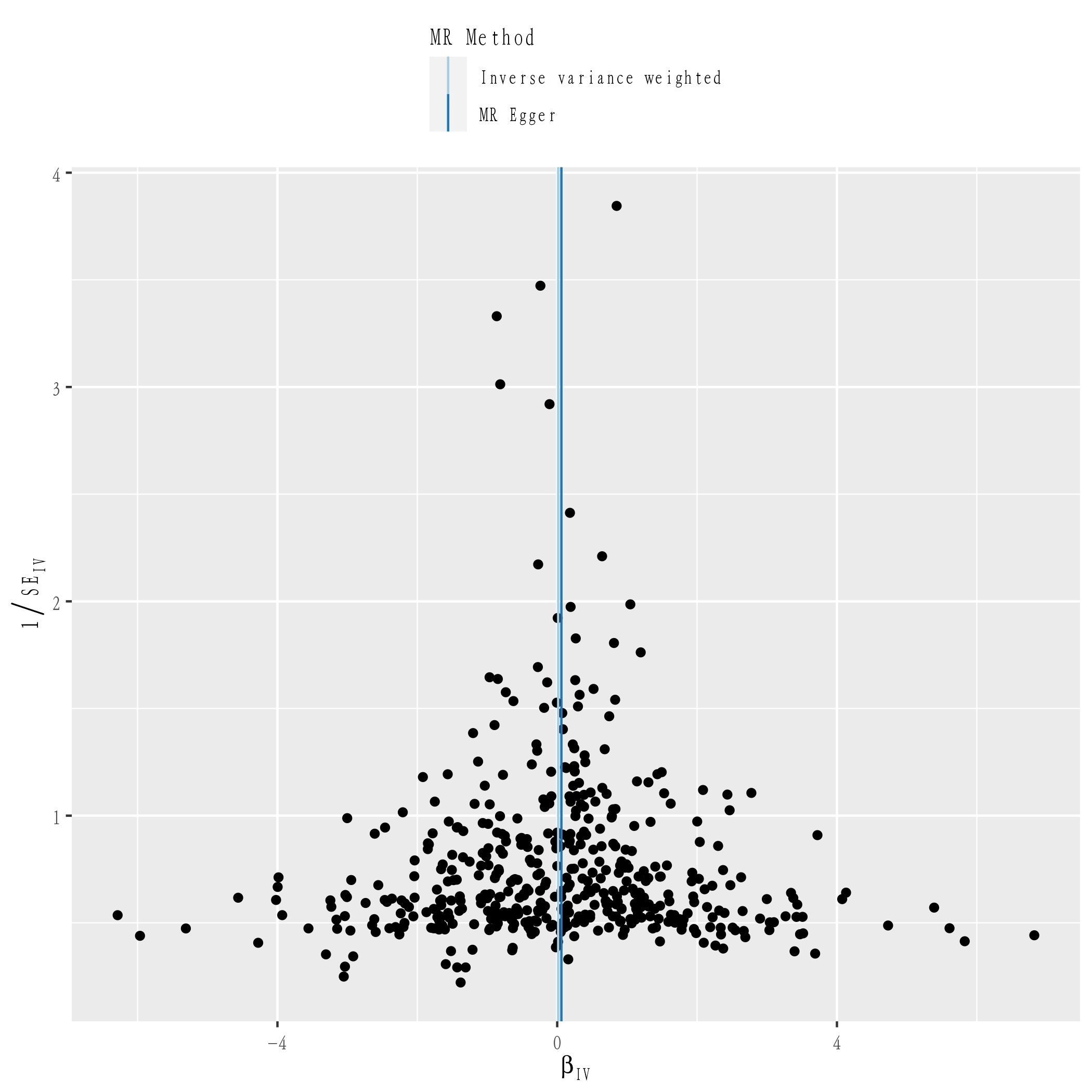

Supplement: Supplementary file 1 [file Data_Sheet_1.ZIP › Supplementary Figure 40.tif]

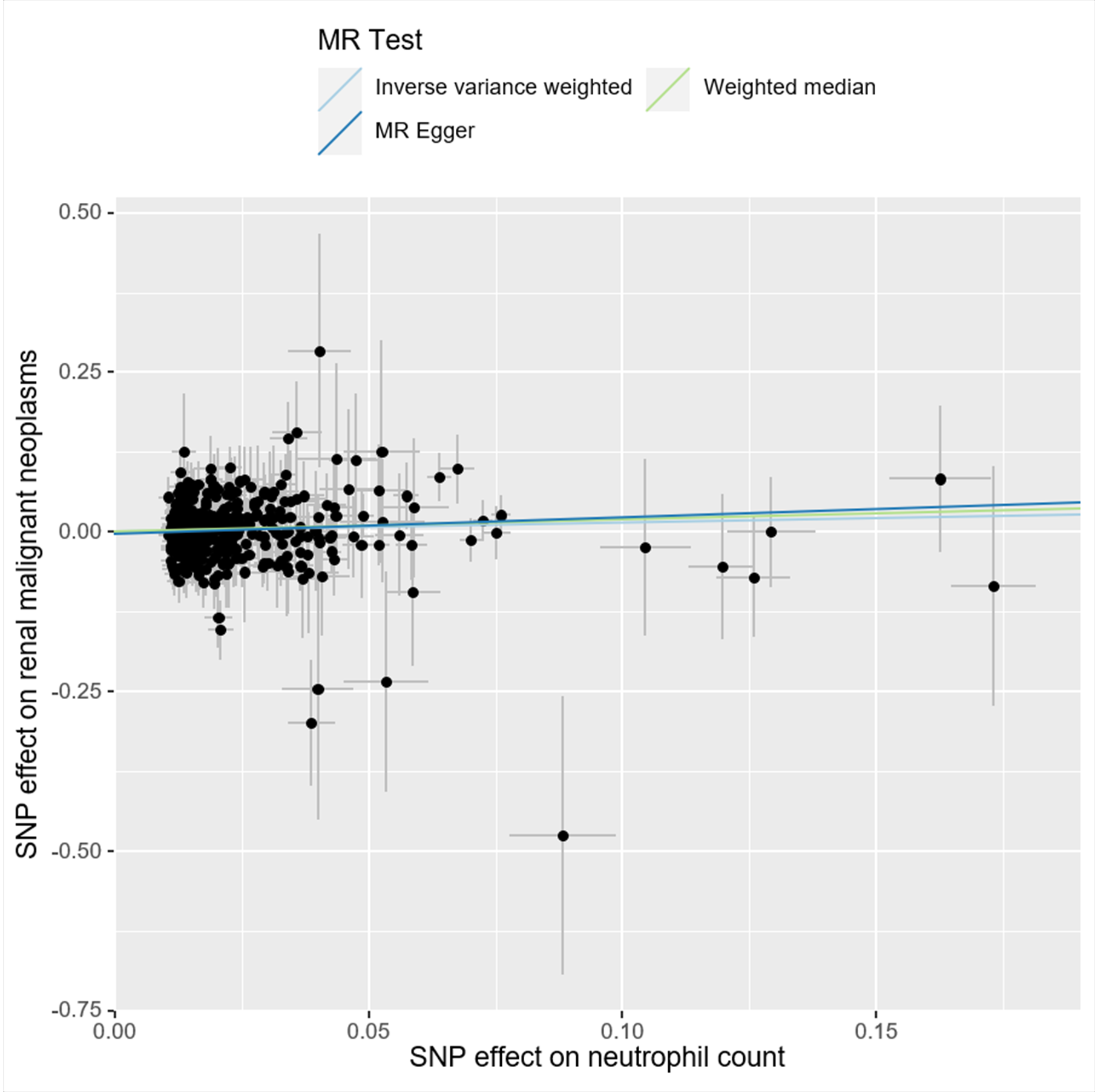

Supplement: Supplementary file 1 [file Data_Sheet_1.ZIP › Supplementary Figure 5.tif]

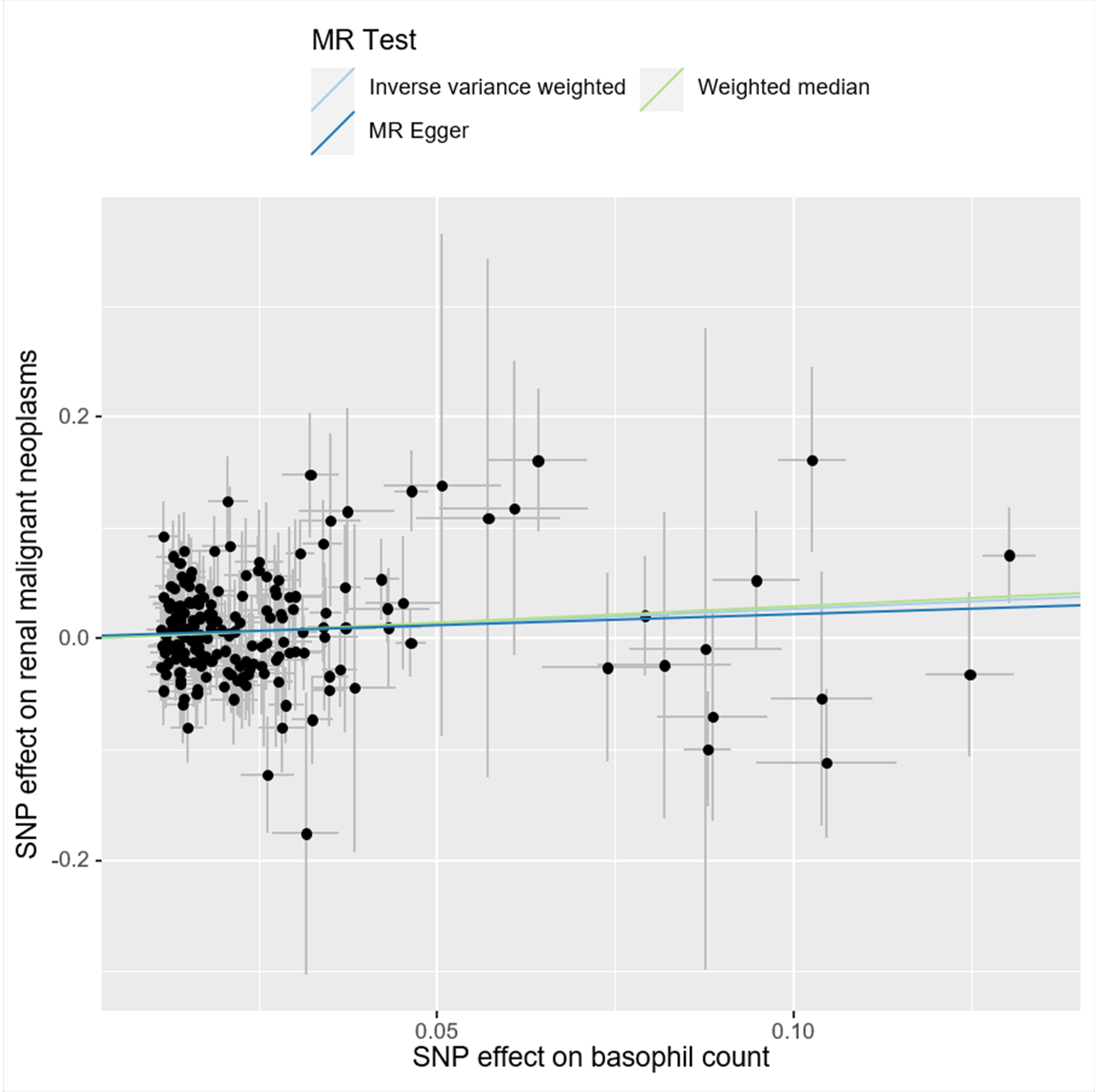

Supplement: Supplementary file 1 [file Data_Sheet_1.ZIP › Supplementary Figure 6.tif]

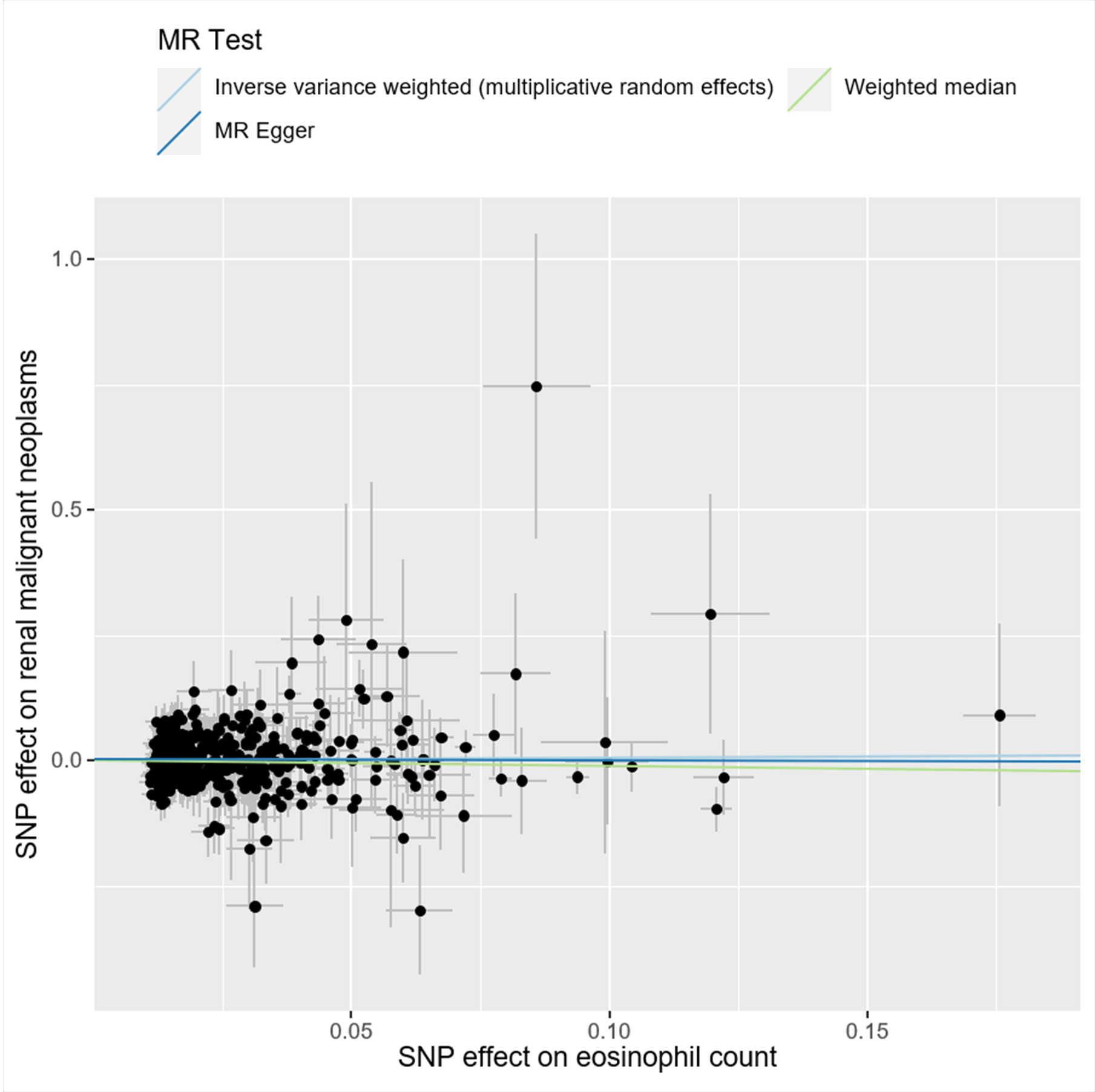

Supplement: Supplementary file 1 [file Data_Sheet_1.ZIP › Supplementary Figure 7.tif]

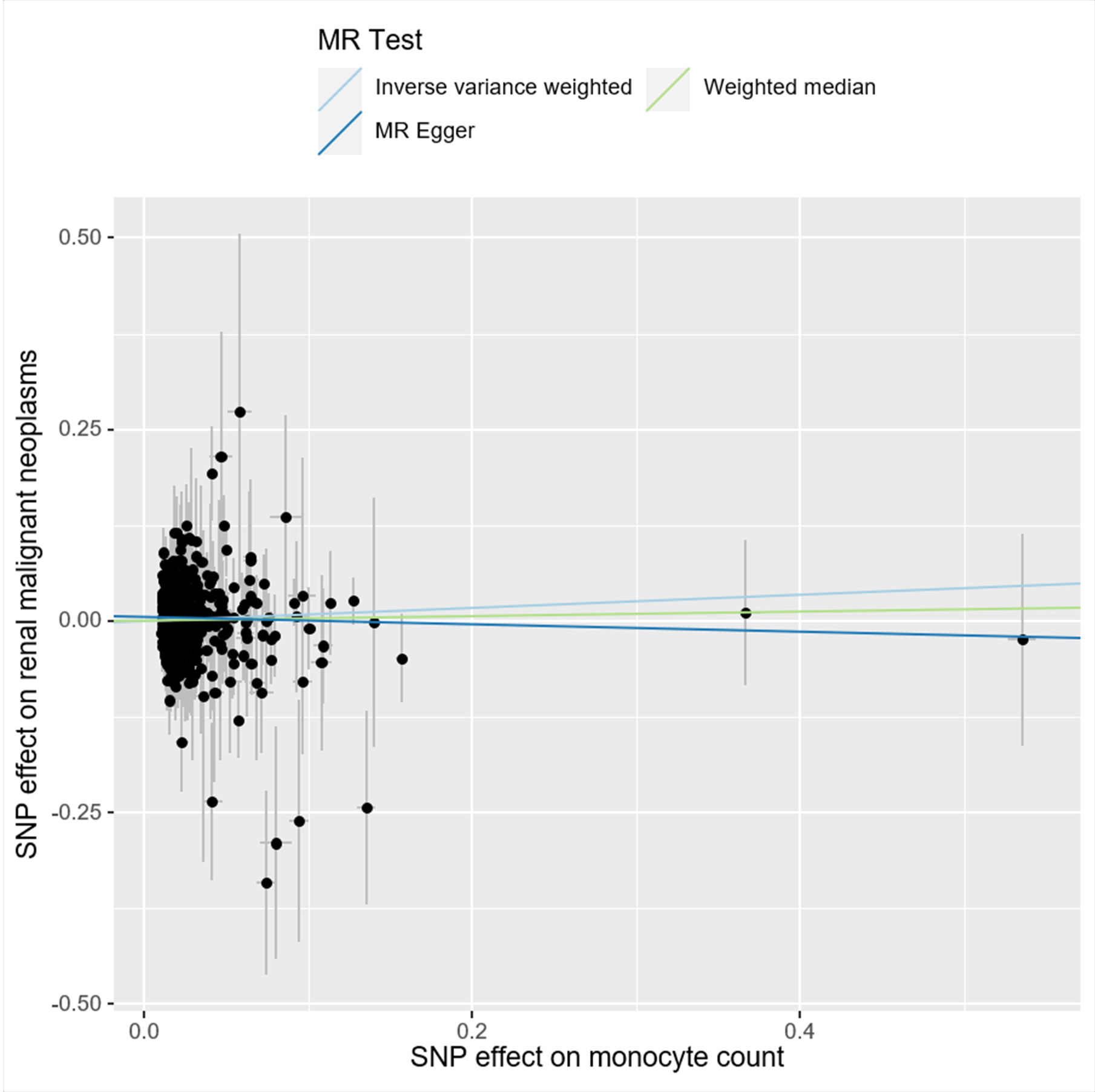

Supplement: Supplementary file 1 [file Data_Sheet_1.ZIP › Supplementary Figure 8.tif]

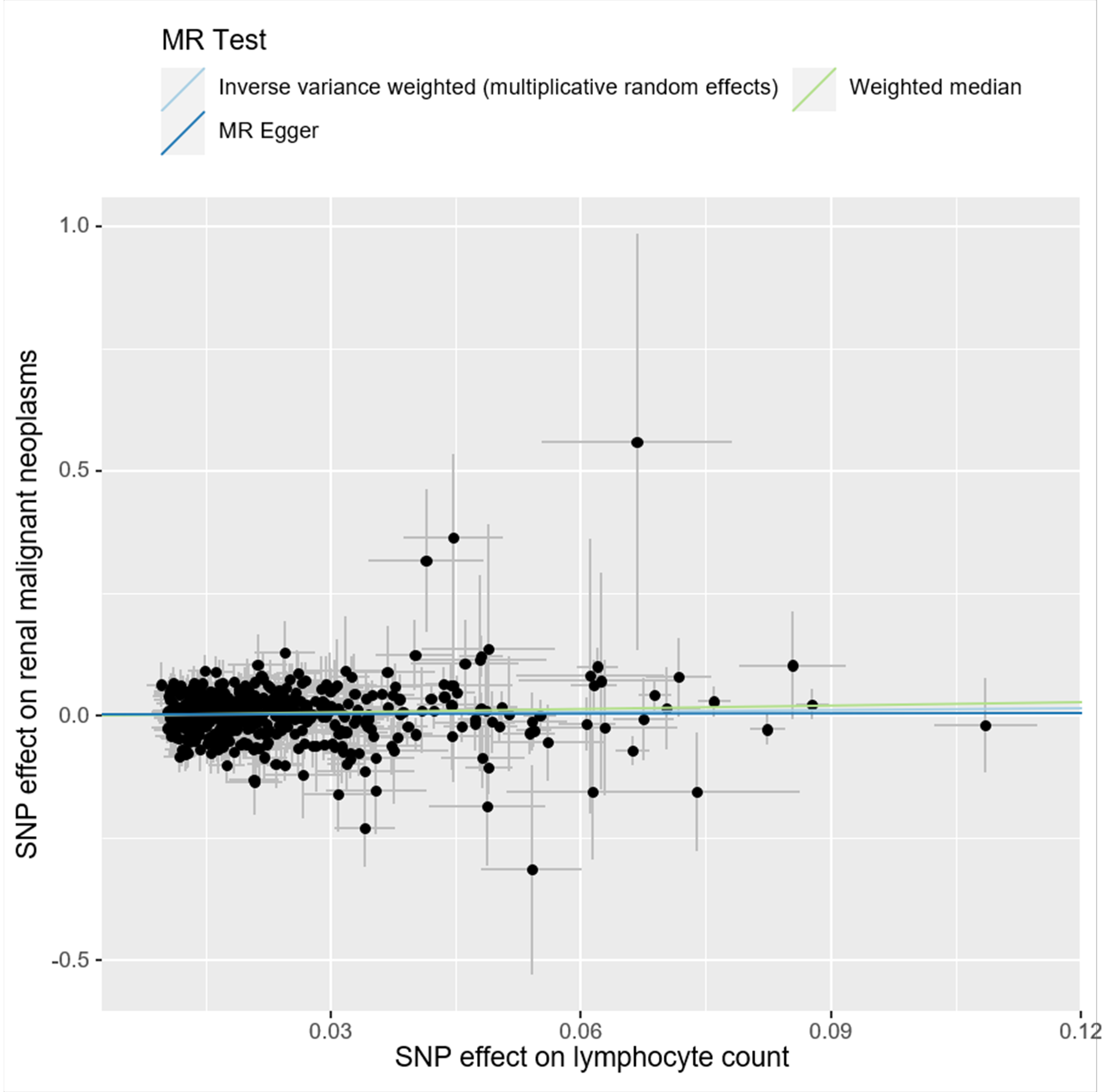

Supplement: Supplementary file 1 [file Data_Sheet_1.ZIP › Supplementary Figure 9.tif]
