# Supplementary material for: Mendelian randomization analysis reveals causal relationships between circulating cell traits and renal disorders
Source: Front Med (Lausanne). 2024 May 17;11:1360868. doi: 10.3389/fmed.2024.1360868 (PMC11140107; doi:10.3389/fmed.2024.1360868)
Supplement: Supplementary file 3 [file Table_2.DOCX]

**Table S2. Sensitivity analyses between circulating cell traits and renal disorders**

| Exposure | Outcome | Heterogeneity | | Pleiotropy | | |
| --- | --- | --- | --- | --- | --- | --- |
|  |  | IVW Q | *P* | | Intercept | *P* |
| White blood cell | IgA nephropathy | 597.16 | 6.84 x10^-6^ | | -0.015 | 0.053 |
| Neutrophil |  | 433.21 | 0.079 | | -0.008 | 0.321 |
| Basophil |  | 215.65 | 0.074 | | 0.016 | 0.116 |
| Eosinophil |  | 511.16 | 0.002 | | 0.004 | 0.575 |
| Monocyte |  | 551.91 | 0.004 | | 0.006 | 0.337 |
| Lymphocyte |  | 359.67 | 0.997 | | -0.009 | 0.202 |
|  |  |  |  | |  |  |
| White blood cell | Renal malignant neoplasms | 495.71 | 0.086 | | 0.001 | 0.689 |
| Neutrophil |  | 416.81 | 0.196 | | 0.004 | 0.423 |
| Basophil |  | 219.54 | 0.052 | | 0.002 | 0.708 |
| Eosinophil |  | 492.55 | 0.008 | | 0.004 | 0.505 |
| Monocyte |  | 518.08 | 0.051 | | 0.005 | 0.079 |
| Lymphocyte |  | 540.19 | 0.012 | | 0.003 | 0.435 |
|  |  |  |  | |  |  |
| White blood cell | Hypertensive nephropathy | 480.06 | 0.192 | | 0.002 | 0.763 |
| Neutrophil |  | 406.10 | 0.314 | | 0.006 | 0.307 |
| Basophil |  | 176.42 | 0.670 | | 0.002 | 0.758 |
| Eosinophil |  | 453.06 | 0.151 | | -0.0005 | 0.923 |
| Monocyte |  | 461.37 | 0.565 | | 0.005 | 0.193 |
| Lymphocyte |  | 417.29 | 0.958 | | -0.001 | 0.783 |
|  |  |  |  | |  |  |
| White blood cell | Diabetic nephropathy | 343.57 | 0.993 | | 0.005 | 0.080 |
| Neutrophil |  | 295.01 | 0.990 | | 0.005 | 0.073 |
| Basophil |  | 267.76 | 5.40 x10^-5^ | | -0.001 | 0.754 |
| Eosinophil |  | 565.97 | 2.43 x10^-6^ | | 0.001 | 0.646 |
| Monocyte |  | 589.06 | 9.06 x10^-5^ | | 0.0009 | 0.705 |
| Lymphocyte |  | 559.76 | 0.002 | | -0.001 | 0.656 |

IVW: inverse-variance weighted; *P_FDR_*: *P* value after false discovery rate correction.

**Supplementary Figure legends**

**Supplementary Figure 1** Scatter plot of SNP-neutrophil count and SNP-IgA nephropathy association.

**Supplementary Figure 2** Scatter plot of SNP-basophil count and SNP-IgA nephropathy association.

**Supplementary Figure 3** Scatter plot of SNP-eosinophil count and SNP-IgA nephropathy association.

**Supplementary Figure 4** Scatter plot of SNP-monocyte count and SNP-IgA nephropathy association.

**Supplementary Figure 5** Scatter plot of SNP-neutrophil count and SNP-renal malignant neoplasms association.

**Supplementary Figure 6** Scatter plot of SNP-basophil count and SNP-renal malignant neoplasms association.

**Supplementary Figure 7** Scatter plot of SNP-eosinophil count and SNP-renal malignant neoplasms association.

**Supplementary Figure 8** Scatter plot of SNP-monocyte count and SNP-renal malignant neoplasms association.

**Supplementary Figure 9** Scatter plot of SNP-lymphocyte count and SNP-renal malignant neoplasms association.

**Supplementary Figure 10** Scatter plot of SNP-white blood cell count and SNP-hypertensive nephropathy association.

**Supplementary Figure 11** Scatter plot of SNP-neutrophil count and SNP-hypertensive nephropathy association.

**Supplementary Figure 12** Scatter plot of SNP-basophil count and SNP-hypertensive nephropathy association.

**Supplementary Figure 13** Scatter plot of SNP-eosinophil count and SNP-hypertensive nephropathy association.

**Supplementary Figure 14** Scatter plot of SNP-monocyte count and SNP-hypertensive nephropathy association.

**Supplementary Figure 15** Scatter plot of SNP-lymphocyte count and SNP-hypertensive nephropathy association.

**Supplementary Figure 16** Scatter plot of SNP-white blood cell count and SNP-diabetic nephropathy association.

**Supplementary Figure 17** Scatter plot of SNP-neutrophil count and SNP-diabetic nephropathy association.

**Supplementary Figure 18** Scatter plot of SNP-basophil count and SNP-diabetic nephropathy association.

**Supplementary Figure 19** Scatter plot of SNP-monocyte count and SNP-diabetic nephropathy association.

**Supplementary Figure 20** Scatter plot of SNP-lymphocyte count and SNP-diabetic nephropathy association.

**Supplementary Figure 21** Funnel plot of neutrophil count and IgA nephropathy.

**Supplementary Figure 22** Funnel plot of basophil count and IgA nephropathy.

**Supplementary Figure 23** Funnel plot of eosinophil count and IgA nephropathy.

**Supplementary Figure 24** Funnel plot of monocyte count and IgA nephropathy.

**Supplementary Figure 25** Funnel plot of neutrophil count and renal malignant neoplasms.

**Supplementary Figure 26** Funnel plot of basophil count and renal malignant neoplasms.

**Supplementary Figure 27** Funnel plot of eosinophil count and renal malignant neoplasms.

**Supplementary Figure 28** Funnel plot of monocyte count and renal malignant neoplasms.

**Supplementary Figure 29** Funnel plot of lymphocyte count and renal malignant neoplasms.

**Supplementary Figure 30** Funnel plot of white blood cell count and hypertensive nephropathy.

**Supplementary Figure 31** Funnel plot of neutrophil count and hypertensive nephropathy.

**Supplementary Figure 32** Funnel plot of basophil count and hypertensive nephropathy.

**Supplementary Figure 33** Funnel plot of eosinophil count and hypertensive nephropathy.

**Supplementary Figure 34** Funnel plot of monocyte count and hypertensive nephropathy.

**Supplementary Figure 35** Funnel plot of lymphocyte count and hypertensive nephropathy.

**Supplementary Figure 36** Funnel plot of white blood cell count and diabetic nephropathy.

**Supplementary Figure 37** Funnel plot of neutrophil count and diabetic nephropathy.

**Supplementary Figure 38** Funnel plot of basophil count and diabetic nephropathy.

**Supplementary Figure 39** Funnel plot of monocyte count and diabetic nephropathy.

**Supplementary Figure 40** Funnel plot of lymphocyte count and diabetic nephropathy.
